# Strategies to promote liver fibrosis amelioration with involvement of restorative macrophages

**DOI:** 10.3389/fimmu.2026.1792423

**Published:** 2026-05-12

**Authors:** Jorge Benjamin Aquino

**Affiliations:** 1 Developmental Biology & Regenerative Medicine Laboratory, Instituto de Investigaciones en Medicina Traslacional, CONICET-Universidad Austral, Derqui, Pilar, Buenos Aires, Argentina; 2 Facultad de Ciencias Biomédicas, Universidad Austral, Derqui, Pilar, Buenos Aires, Argentina; 3 Facultad de Ingeniería, Universidad Austral, Derqui, Pilar, Buenos Aires, Argentina

**Keywords:** antifibrotic therapy, immunometabolic reprogramming, liver fibrosis, macrophage plasticity, restorative macrophages, macrophages, inflammation, fibrosis resolution

## Abstract

Chronic liver diseases represent a major global health burden, with fibrosis as the common pathological outcome of sustained hepatic injury. Once considered irreversible, liver fibrosis is now recognized as a dynamic and potentially reversible process driven by complex cellular and molecular interactions within the hepatic microenvironment. Among these, hepatic macrophages have emerged as central regulators of both fibrogenesis and fibrosis resolution due to their remarkable phenotypic and functional plasticity. This review integrate experimental, translational, and emerging clinical evidence to propose macrophage reprogramming as a unifying therapeutic framework for liver fibrosis. A broad spectrum of intervention strategies -including gene modulation, pharmacological agents, immunometabolic reprogramming, nanotechnology-based delivery systems, and cell-based therapies- converges on promoting restorative macrophage phenotypes across toxic, metabolic, cholestatic, and inflammatory liver diseases. Particular emphasis is placed on key signaling and metabolic circuits -such as NF-κB, STAT1/3/6, PPARα/γ, AMPK, mitochondrial function, and autophagy- that collectively govern macrophage fate and function. The context-dependent nature of macrophage responses is highlighted, underscoring critical differences between toxic injury models (e.g., CCl_4_) and chronic metabolic conditions such as MASH, where macrophage heterogeneity and immunometabolic dysregulation impose additional therapeutic challenges. Emerging clinical data indicate that many antifibrotic strategies -despite distinct primary targets- converge on shared pathways of macrophage modulation, reinforcing their role as integrative hubs linking inflammation, metabolism, and tissue repair. Collectively, current findings indicate that durable fibrosis regression is unlikely to be achieved through single-target interventions. Instead, effective therapeutic strategies will require coordinated, temporally defined modulation of macrophages alongside other hepatic cells populations. Elucidation of the hierarchy and timing of macrophage-driven repair processes will be essential for the rational design of next-generation antifibrotic interventions with improved clinical efficacy.

## Introduction

The liver exhibits a remarkable regenerative capacity, making it an excellent model for studying endogenous mechanisms of tissue repair. Liver fibrosis arises from a dynamic imbalance between extracellular matrix (ECM) deposition and degradation, driven by persistent injury, chronic inflammation, and dysregulated repair processes. In chronic liver diseases where the underlying cause cannot be eliminated, effective therapeutic options remain limited, and liver transplantation continues to represent the only curative intervention.

Fibrosis resolution is a coordinated process characterized by extracellular matrix degradation (associated with increased metalloproteinase expression), myofibroblast inactivation or apoptosis (reflected by reduced collagen and α-smooth muscle actin protein expression), and restoration of normal tissue architecture and function.

Macrophages play a central, context-dependent role throughout all stages of liver fibrosis, from initiation to resolution. Historically, macrophage activation has been simplified into pro-inflammatory (M1-like) and anti-inflammatory (M2-like) phenotypes (1). However, advances in single-cell transcriptomics and fate-mapping approaches have challenged this binary framework, revealing a highly heterogeneous and plastic macrophage landscape. Distinct populations -including resident Kupffer cells (KCs), monocyte-derived KCs (Mo-KCs), and lipid-associated macrophages (LAMs)- exhibit specialized and dynamic transcriptional programs tightly regulated by microenvironmental cues.

Importantly, macrophages are now recognized not only as drivers of fibrogenesis but also as key mediators of fibrosis resolution. **Restorative (or pro-resolving) macrophages** contribute to extracellular matrix degradation, myofibroblast inactivation, and tissue remodeling. They secrete growth factors such as insulin-like growth factor-I (IGF-I) and hepatocyte growth factor (HGF), which support tissue repair and regeneration (1–4). These functions are governed by coordinated immunometabolic reprogramming, involving shifts in signaling pathways (e.g., NF-κB, STATs, PPARs), metabolic states (glycolysis versus oxidative phosphorylation), and intercellular communication networks. Across multiple experimental models, macrophages with these properties have been identified as critical mediators of fibrosis resolution.

In this context, macrophage-targeted therapeutic strategies have emerged as a promising approach to promote fibrosis regression (5). These include modulation of intracellular signaling pathways, metabolic reprogramming, nanotechnology-based delivery systems, and cell-based therapies. Some strategies aim to enhance phagocytic activity and antigen clearance (1, 3, 6). In particular, macrophage-mediated efferocytosis -the immunologically silent clearance of apoptotic cells have shown to promote hepatic stellate cell (HSC) deactivation (7) and to induce interleukin-10 (IL-10) production, which further suppresses HSC profibrotic activity via IL-10 receptor signaling.

In this review, we provide an updated and integrative framework of macrophage-driven fibrosis resolution, moving beyond the classical M1/M2 paradigm. We first define the heterogeneity and functional states of liver macrophages, then discuss key mechanistic pathways underlying restorative macrophage activity, and finally examine emerging therapeutic strategies and their translational potential, including limitations, risks, and context-dependent effects across different etiologies of liver fibrosis.

## Liver macrophage heterogeneity and plasticity in fibrosis

Recent advances in single-cell and spatial transcriptomics in mouse models have challenged the classical M1/M2 dichotomy, revealing a highly heterogeneous and dynamic spectrum of liver macrophage states. In metabolic-associated steatotic liver disease (MASLD) -which encompasses a range of conditions spectrum of disease states ranging from simple steatosis to metabolic dysfunction-associated steatohepatitis (MASH)- KCs, Mo-KCs, and LAMs exhibit context-dependent transcriptional programs that cannot be captured by binary classification systems (8, 9).

Remmerie et al. (8) demonstrated that the resident KCs progressively decline during disease progression and are replaced by distinct subpopulations derived from bone marrow monocytes. Among these, LAMs are characterized by expression of TREM2 and high levels of Spp1 (osteopontin). These cells localize to fibrotic regions and act as key contributors to liver fibrogenesis. Similarly, Daemen et al. (9), using high fat diet model of MASH, reported replacement by KCs by Mo-KCs and identified two subsets of monocyte-derived TREM2^+^ macrophages: one with high Spp1 expression (LAMs), and another with elevated Cx3Cr1/Ccr2 expression (termed C-LAMs). C-LAMs may modulate fibrogenesis and likely represent transitional states between infiltrating monocytes and differentiated KCs or LAMs. Both LAMs and C-LAMs preferentially localized to hepatic regions enriched in HSCs.

Consistent with these findings, Ganguli et al. (10) identified Mo-KCs and LAMs as the predominant macrophage populations in MASH. Notably, during disease regression, fibrosis resolution was associated not with the emergence of new macrophage subsets but with a shift in population dynamics. While Mo-KCs predominate during active disease, LAMs become dominant during regression, maintaining TREM2 expression. These TREM2^+^ macrophages play a critical role in limiting fibrosis progression and promoting resolution of inflammation and scarring through enhanced phagocytosis, lipid handling, and collagen turnover.

De Ponti et al. (11) further showed that LAMs also emerge in other liver injury models, including acetaminophen (APAP)-induced acute injury and carbon tetrachloride (CCl_4_)-induced fibrosis. In these contexts, resident KCs persist but acquire a LAM-like phenotype. Similar LAM-like KCs were identified in **human** liver samples from patients with APAP overdose. Efferocytosis of bone marrow-derived macrophages (BMDM) was found to upregulate LAM-associated gene expression, and TREM2 overexpression in both LAM and LAM-like KCs was required for tissue repair, partly through enhanced efferocytic capacity in the CCl_4_ model.

Collectively, these findings support a model in which macrophages exist along a functional continuum shaped by environmental cues such as cytokines, metabolites, dying cells, and microbial signals. Within this spectrum, restorative macrophages represent a functional state characterized by enhanced efferocytosis, secretion of matrix-degrading enzymes (e.g., MMPs), anti-inflammatory cytokine production, and promotion of tissue repair.

These insights underscore that macrophage phenotypes are not fixed entities but dynamic ad plastic states amenable to therapeutic reprogramming. A deeper understanding of this heterogeneity will be critical for the development of targeted strategies aimed at promoting fibrosis resolution.

## Mechanistic basis of restorative macrophages

**Figure 1:**
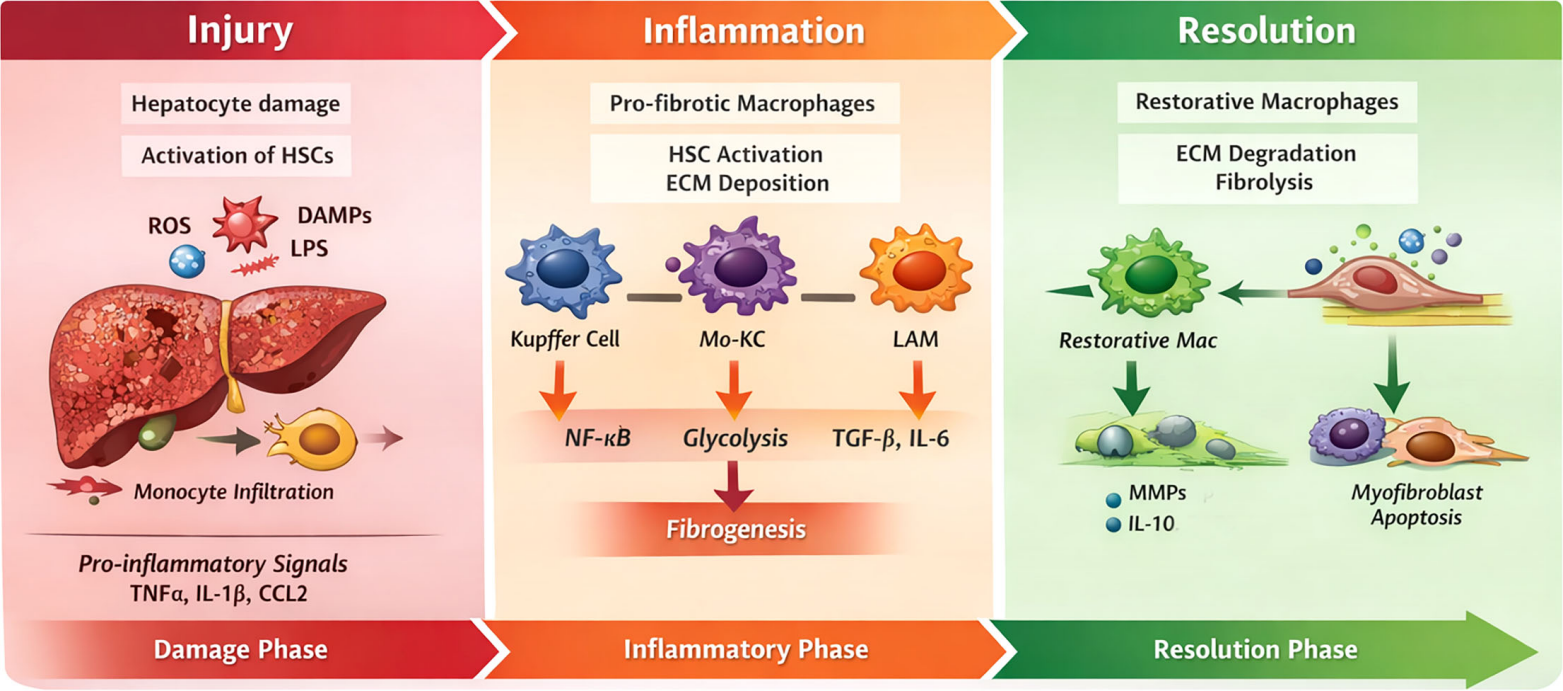
Liver fibrosis and resolution. Schematic showing main different roles played by macrophages in temporal phases of injury, inflammation and resolution and main signaling events involved.

A simple overview of the key events underlying fibrogenesis onset and the transition from pro-fibrotic to restorative macrophages is presented in **Figure 1**.

Using a CCl_4_-induced mouse model, Zhang et al. (6) demonstrated that low levels of reactive oxygen species (ROS) generated during fibrosis regression induce expression of the transient receptor potential melastatin 2 (TRPM2) channels in macrophages. Activation of TRPM2 promotes acquisition of a restorative phenotype and enhances macrophage phagocytic activity. Mechanistically, this process involves increased calcium-dependent mitochondrial fission in TRPM2^+^ macrophages, which augments efferocytosis and facilitates the transition from a pro-fibrotic to a pro-resolutive state.

Targeting oxidative stress and inflammatory signaling alone may be sufficient to drive pro-fibrotic macrophage reprogramming into a reparative phenotype. **Inhibition of NF-κB** -a key transcription factor active in pro-fibrotic macrophages-, in RAW264.7 cells reduces inducible nitric oxide synthase (iNOS) expression and dampens inflammatory responses (12, 13). Transcriptomic analyses further revealed that NF-κB–dependent inflammatory genes are major downstream targets of the histone methyltransferase MLL4 (14), which directly interacts with the NF-κB subunit p65 and is required for its full transcriptional activity. Accordingly, myeloid-specific deletion of Mll4 nearly abolishes MASH pathology and promotes a shift of hepatic macrophages toward a restorative phenotype.

Consistent with these findings, a similar phenotypic switch was observed in RAW264.7 cells cultured under hypoxic conditions, where inactivation of the NF-κB/HIF-1α axis favors a reparative state (15). He et al. (16) further demonstrated that attenuation of NF-κB signaling is mediated by upregulation of cylindromatosis (CYLD), a negative regulator of inflammatory signaling. In line with this, Lee et al. (17) showed that Sirtuin 6 (SIRT6) deficiency in BMDMs enhances NF-κB activation, leading to increased IL-6 production, subsequent STAT3 activation, and amplification of NF-κB signaling, ultimately reinforcing a profibrotic macrophage phenotype.

### Metabolic reprogramming as a central driver of macrophage fate

Several chemokines upregulated during fibrogenesis -including CCL2, CCL5, CCL20, and CXCL10- induce expression of the N-glycosylated tetraspanin TM4SF5 in macrophages (18). Kim et al. (19) demonstrated that pro-fibrotic-polarized **human** macrophages ectopically expressing TM4SF5 secrete IL-6, which stimulates TM4SF5^+^ hepatocytes to release CCL20 and CXCL10. Conditioned medium from these hepatocytes further enhances endogenous TM4SF5 expression in macrophages and upregulates pro-fibrotic macrophage markers. Mechanistically, TM4SF5 likely interacts with GLUT1 at the macrophage surface, promoting glucose uptake and glycolytic flux, thereby reinforcing pro-fibrotic polarization.

Interestingly, chronic exposure of pro-fibrotic macrophages to CCL20 induces expression of pro-resolutive macrophage markers and promotes a metabolic shift toward increased oxidative phosphorylation, suggesting that TM4SF5^+^ hepatocytes may also contribute to macrophage repolarization. Consistently, TM4SF5^-/-^ mice are protected from liver fibrosis in MASH models, and BMDMs from these mice display reduced pro-fibrotic polarization and enhanced expression of restorative markers. These findings support the concept that metabolic reprogramming alone may be sufficient to dictate macrophage fate. In this framework, pro-fibrotic macrophages are characterized by enhanced glycolysis and reduced tricarboxylic acid (TCA) cycle activity (20), whereas restorative macrophages rely predominantly on mitochondrial metabolism, including fatty acid oxidation and oxidative phosphorylation (21).

Pyruvate kinase M2 (PKM2) emerges as a key metabolic regulator linking glycolysis and oxidative metabolism in macrophages (22, 23). Its deacetylation reduces lactate production and attenuates liver fibrosis (24), whereas PKM2 activation and decreased ubiquitination enhance glycolysis flux and promote pro-fibrotic polarization (25). Similarly, inhibition of chitinase 1 (CHIT1) using OATD-01 reduces glucose uptake, restores ATP levels, suppresses IL-1β secretion, and rebalances macrophage metabolism, further underscoring the central role of metabolic control in fibrosis regression (26).

### Mitochondrial signaling and autophagy

Given the high mitochondrial content of hepatocytes, mitochondrial dysfunction and the release of mitochondrial DNA (mtDNA) act as potent danger signals in the fibrotic liver (27). Conversely, improved mitochondrial function -including enhanced oxidative phosphorylation and mitochondrial biogenesis- has been consistently associated with macrophage reprogramming and fibrosis resolution (28–31). In this context, Lin et al. (32) showed that SGLT2 inhibition targets PFKFB3, a key glycolytic enzyme, thereby promoting a metabolic shift from glycolysis to oxidative phosphorylation and favoring restorative macrophage polarization.

Elegant proof of concept for mitochondria-driven macrophage reprogramming was provided by Che et al. (27), who developed a photosensitizer-loaded, peptide-decorated nanosystem selectively targeting integrin αVβ3-expressing HSCs. By incorporating mitochondrial targeting sequences, this platform enabled precise mitochondrial localization. Upon light activation, localized mitochondrial stress induced outer membrane permeabilization and release of mtDNA into the fibrotic microenvironment. This mtDNA was sensed by macrophages via the cGAS–STING pathway, triggering immune-stimulatory transcriptional programs and promoting their conversion toward a restorative phenotype. This mitochondria-mediated HSC–macrophage crosstalk ultimately facilitated fibrosis reversal and restoration of hepatic homeostasis.

AMP-activated protein kinase (AMPK), a master regulator of cellular energy homeostasis, plays a central role in this process (33–36). Its activation suppresses mTOR and NF-κB signaling while promoting mitochondrial biogenesis via peroxisome proliferator-activated receptor gamma coactivator-1α (PGC-1α), mechanisms collectively associated with restorative macrophage polarization (34, 37).

Autophagy, frequently impaired during fibrogenesis, is essential for mitochondrial quality control and metabolic homeostasis. Its induction promotes a shift toward a restorative macrophage phenotype, whereas proinflammatory stimuli such as LPS suppress autophagic flux and favor pro-fibrotic polarization (34, 38–40). Impaired autophagy also disrupts lipid handling in hepatocytes, leading to mitochondrial dysfunction and increased ROS production. Notably, several antifibrotic interventions -including traditional Chinese medicinal (TCM) compounds and pharmaceutical agents such as metformin, ***glucagon-like peptide-1 receptor agonists (GLP-1RAs)*** or ***SGLT2 inhibitors (SGLT2is)***- promote restorative macrophage polarization, at least in part, through activation of AMPK-dependent pathways and enhance hepatic autophagy (34, 39, 41–44).

### Other pathways in the transcriptional regulation of restorative macrophages

Downregulation or inhibition of additional signaling pathways implicated in pro-fibrotic polarization has also been shown to favor the acquisition of a restorative phenotype in macrophages. For instance, modulation of the PI3K/AKT/mTOR pathway ameliorates liver fibrosis through macrophage-HSC crosstalk, in association with enhanced restorative macrophage polarization (23, 45, 46). Moreover, inhibition of ERK/AKT, IRF5, JAK1/JAK2-STAT1, MAP kinase and Notch1 signaling pathways consistently promotes restorative macrophages polarization and attenuates fibrogenic responses (47–55).

Interestingly, activation of the MEK–ERK–c-MYC axis in splenic macrophages has also been reported to promote a restorative phenotype and reduce liver fibrosis (56). In line with this, Ramachandran et al. (1) showed that phagocytosis-driven ERK activation recapitulates restorative macrophage features *in vitro* and accelerates fibrosis resolution *in vivo*, highlighting the context-dependent role of ERK signaling.

STAT pathways are central regulators of macrophage fate. STAT3 activation is generally associated with restorative polarization and attenuation of fibrosis, whereas STAT1 signaling correlates with pro-fibrotic phenotypes and cirrhosis (47, 57, 58). Accordingly, an increased phosphorylated STAT3 (p-STAT3) to phosphorylated STAT1 (p-STAT1) ratio favors acquisition of a restorative phenotype (59). Campana et al. (60) further demonstrated that efferocytosis activates a STAT3-IL-10 autocrine/paracrine loop that preserves macrophage scavenging capacity and promotes restorative polarization. However, STAT3 signaling can also support pro-fibrotic polarization under specific inflammatory conditions, underscoring its highly context-dependent function (52, 61, 62).

STAT6 represents another key driver of restorative polarization (49, 63, 64). Interleukin-4 (IL-4) induces this phenotype via IL-4 receptor α (IL-4Rα) and subsequent STAT6 activation. Mechanistically, IL-4 and IL-13 were shown to activate STAT6 in macrophages, leading to overexpression of the fibrolytic enzyme MMP-12 (65). Notably, disruption of IL-4Rα signaling has yielded context-dependent effects: while genetic deletion reduces macrophage populations and ameliorates fibrosis, antisense-mediated inhibition during regression impairs restorative polarization and delays fibrosis resolution (65). Conversely, forkhead box O1 (FoxO1) antagonizes STAT6 signaling, promoting pro-fibrotic macrophage polarization and sustaining inflammation in MASH (66).

STAT6 further regulates macrophage fate through downstream activation of nuclear receptors, including PPARγ and Nrf2 (67). PPARγ is required for restorative polarization in experimental liver fibrosis models (68–73) and promotes a phenotypic switch toward anti-inflammatory macrophages in **human** monocytes (74). At the molecular level, PPARγ directly enhances transcription of matrix-remodeling genes such as MMP-10 during IL-4–driven polarization, as demonstrated in RAW264.7 cells (57).

Jansakun et al. (75) reported that BMDMs lacking group VIA calcium-independent phospholipase A_2_ (Pla2g6) display increased PPARγ and C/EBPα expression and upregulation of restorative-associated genes. However, these cells also exhibit elevated IL-6 and CXCL1 production upon LPS stimulation and increased expression of CCR2 and CCR5, indicating a mixed or dysregulated phenotype. *In vivo*, this translated into enhanced recruitment of inflammatory and restorative macrophages, ultimately exacerbating inflammation and fibrosis in MASH models.

PPARα, a key regulator of fatty acid β-oxidation and oxidative phosphorylation, is inversely correlated with MASH severity and positively associated with restorative macrophage abundance in both **human** and experimental models (76, 77). Pharmacological activation of PPARα increases restorative macrophage populations (30), and its upregulation, together with PPARγ, may depend on AMPK activation, linking transcriptional control with metabolic reprogramming (35).

Additional transcription factors further shape macrophage plasticity. CCAAT/enhancer-binding protein α (C/EBPα) promotes restorative polarization in murine macrophages (75, 78). while hepatocyte-specific deletion of C/EBPβ at the time of alcohol cessation induces a pro-resolutive macrophage phenotype during alcohol-associated liver disease, highlighting the importance of hepatocyte–macrophage crosstalk (79).

RORα and KLF4 have also emerged as key regulators of restorative polarization, conferring protection against MASH in murine models (63, 80). In **human** macrophages, reduced SUMOylation activates KLF4, which induces suppressor of cytokine signaling 1 (SOCS1) and inhibits JAK–STAT and TLR4–MyD88 signaling, thereby reducing NF-κB and MAP kinase activity and promoting restorative polarization (81–85).

Ubiquitin-mediated regulatory pathways also contribute to macrophage reprogramming. Moreno-Lanceta et al. (4) showed that restoration of RNF41 expression in macrophages alleviates liver fibrosis, reduces liver injury, and enhances regeneration through induction of IGF-I, whereas its loss exacerbates disease. Similarly, the E3 ubiquitin ligase TRIM38 promotes restorative polarization by stabilizing heat shock protein family A member 5 (HSPA5) via K63-linked ubiquitination (86).

Together, these pathways converge on a coordinated regulatory network in which STAT signaling, nuclear receptors (PPARs), AMPK-dependent metabolic control, and NF-κB–driven inflammatory programs dynamically interact to determine macrophage fate along the pro-fibrotic–restorative spectrum.

Collectively, these findings highlight the complexity of transcriptional networks governing macrophage plasticity. Coordinated modulation of STAT signaling, nuclear receptors, metabolic regulators, and ubiquitin-mediated pathways emerge as a central mechanism driving restorative macrophage polarization. This growing mechanistic framework provides a strong basis for the rational design of therapeutic strategies aimed at reprogramming macrophages to restore tissue homeostasis and promote fibrosis resolution.

## Strategies inducing restorative macrophages in liver fibrosis

A common strategy to ameliorate liver fibrosis involves counteracting disease-associated alterations in gene expression, as well as dysregulated metabolic and signaling pathways that drive fibrogenesis. Numerous experimental studies -primarily conducted in murine models- have demonstrated that modulation of individual genes, through genetic deletion, pharmacological inhibition, or targeted overexpression, can be sufficient to attenuate or even reverse fibrosis, largely by reshaping macrophage phenotype and function.

### CCl_4_ models

Chronic CCl_4_-induced liver injury is one of the most widely used experimental systems to investigate restorative macrophages. Studies targeting genes upregulated in these models are discussed first.

Bansal et al. (5) showed that components of the Notch signaling pathway -key mediators of *cell-cell communication***-** are upregulated in HSCs, the principal effectors of fibrogenesis, as well as in pro-fibrotic macrophages in both murine and **human** livers. Pharmacological inhibition of Notch signaling using avagacestat, a selective γ-secretase inhibitor, attenuated fibrosis and increased the abundance of pro-resolutive macrophages. Avagacestat also promoted deactivation of human LX2 cells, an established HSC cell line. Consistently, He et al. (16) demonstrated that myeloid-specific deletion of RBP-J, the nuclear effector of Notch signaling, reduces liver fibrosis, likely through up-regulation of cylindromatosis (CYLD), a negative regulator of NF-κB signaling in macrophages.

Importantly, some interventions induce a shift toward a pro-resolutive macrophage phenotype *indirectly*, by targeting genes primarily expressed in other cell types. For example, Wehr et al. (87) analyzed liver fibrogenesis in CXCR6^-/-^ mice. C-X-C chemokine receptor 6 (CXCR6) is mainly expressed in natural killer (NK) cells, whereas its ligand CXCL16 is expressed in macrophages and endothelial cells, and both are also upregulated in **patients** with chronic liver disease. In this model, fibrosis amelioration appeared to depend on effects on NK-cells rather than direct modulation of macrophages, although macrophage phenotypes were not extensively characterized. A similar protective effect was observed in a MASH model, supporting the relevance of intercellular crosstalk in shaping macrophage function.

Yang et al. (88) inhibited fibroblast activation protein (FAP), which is expressed in a subset of myofibroblasts, including HSCs, and observed attenuation of parenchymal liver fibrosis together with increased hepatocyte proliferation. Partial polarization of macrophages toward a restorative phenotype -characterized by downregulation of pro-inflammatory markers without changes in Arg1 expression- was observed during fibrosis progression but not during regression. Consistently, FAP inhibition was effective in limiting fibrogenesis but did not promote fibrosis resolution. Using a similar approach in a biliary fibrosis model (Mdr2^-/-^ mice), FAP inhibition also improved liver fibrosis although macrophages displayed a mixed phenotype, with upregulation of both pro-inflammatory and tolerogenic markers. Importantly, FAP expression was found to be also increased in **patients** with various chronic liver diseases. In complementary *in vitro* experiments, **human** THP-1, monocytes exposed to conditioned media from LX2 cells pretreated with a FAP inhibitor exhibited reduced activation, with downregulation of iNOS, TGFβ1, MMP9 and MMP14, and upregulation of MMP1, along with a trend toward increased IL-10 expression.

Bartneck et al. (89) reported that histidine-rich glycoprotein (HRG), a hepatocyte-derived plasma protein, is upregulated in cirrhotic MASH and hepatitis C virus (HCV) **patients**, as well as in CCl_4_ and MASH mouse models. HRG-deficient mice developed less fibrosis and showed reduced macrophage infiltration, accompanied by a shift toward a restorative phenotype, likely promoting pro-angiogenic and tissue-reparative responses. These findings were further validated in a MASH model.

In other studies, the targeted genes were primarily upregulated *in macrophages* during chronic CCl_4_-induced fibrosis, and their partial or complete expression deficiency -or pharmacological inhibition- was shown to ameliorate fibrosis through mechanisms involving restorative macrophage reprogramming (**Table 1**). These targets include: cannabinoid receptor-1 (CB-1) (90); chemokine receptors such as C-C chemokine receptor 2 (**CCR2**) which is also upregulated in **human** cirrhosis (91, 92), and CCR8, whose inhibition additionally reduced inflammation in a bile-duct ligation (BDL) fibrosis model (93); inflammatory mediators such as cyclooxygenase-2 (COX-2), analyzed in a rat model and known to be also increased in **human** cirrhotic livers, although macrophage phenotypes were less extensively characterized (94); and secreted factors, such as follistatin-like protein 1 (FSTL1), also elevated in **human** cirrhosis, whose myeloid specific deletion also attenuates fibrosis in BDL and MASH models (25).

**Table 1:** Genetic and pharmacological modulation inducing a pro-resolutive phenotype on macrophages in the chronic CCl_4_ mouse model.

Gene/target	Primary function/pathway	Type of modulation	Macrophage signaling pathways involved	Impact on liver fibrosis	Reference
Notch signaling (γ-secretase)	Cell fate determination; macrophage activation	Pharmacological inhibition (Avagacestat)	Non-determined	Reduced collagen deposition	(5)
RBP-J	Nuclear effector of Notch signaling	Myeloid-specific deletion	CYLD–NF-kB inhibition	Attenuated fibrosis	(16)
CXCR6	Controls hepatic NKT cell accumulation at early stages of liver damage	Genetic knock-out	Non-determined	Attenuated fibrosis	(87)
FAP	Expressed on a subset of activated myofibroblasts, associated with macrophage infiltration	Genetic or enzymatic inhibition	Non-determined	Attenuated fibrosis with no effects in fibrosis regression	(88)
HRG	Hepatocyte-derived plasma protein	Genetic knock-out	Non-determined	Decreased fibrosis; pro-angiogenic remodeling	(89)
CB-1	BM-derived monocytes/macrophages activation	Genetic knock-down or pharmacological inhibition	G(α)i/o/RhoA/NF-kB p65 and G(α)i/o/ERK1/2 pathways inhibition	Not determined	(90)
CCR2	GPCR involved in monocyte recruitment	Genetic knock-down/genetic depletion/pharmacological inhibition	NF-*κ*B inhibition (also in THP-1 **human** macrophages). Only In mouse: STAT1 and ERK inhibition	Fibrosis inhibition and regression	(91, 92)
CCR8	Hepatic macrophage recruitment upon injury	Genetic knock-out	Non-determined	Attenuated fibrosis	(93)
COX-2	Key executor of inflammation	Enzymatic inhibition	Activation of PPARγ (hypothesized), and inhibition of MMP-2 and 9	Attenuated fibrosis	(94)
FSTL1	Secreted profibrotic glycoprotein which associate with PKM2 and translocate to the nucleus, promoting glycolysis in macrophages	Genetic knock-out in myeloid cells	TLR-4/NF-kB and STAT1 inhibition, and STAT6 activation, in liver tissue of CCl4, BDL and MASH models. TLR-4/NF-kB inhibition and glycolysis inhibition in BMDMs	Attenuated fibrosis	(25)
IFNAR-1	Innate immune activation	Protein receptor blockade	Increase in pSTAT3/pSTAT1 ratio in monocyte-derived macrophages	Attenuated fibrosis	(59)
Kv1.3	Voltage-gated K^+^ channel	Pharmacological inhibition	STAT1 inhibition and STAT6 activation, as analyzed in RAW264.7 mouse cells	Attenuated fibrosis	(96)
TREM-1	Cell-surface–activating receptor and member of the Ig superfamily that potently amplifies inflammatory responses	Genetic knock-out	Non-determined	Attenuated fibrosis	(95)
MMP-9	ECM degradation enzyme found in macrophages	Targeted overexpression in pro-fibrotic macrophages	Non-determined	Attenuated fibrosis	(97)
IL-22	Anti-inflammatory IL-10 family cytokine	Treatment with the recombinant protein	Upregulation of STAT3 and downregulation of Erk and Akt, in primary mouse cultured fibrotic macrophages and **human** histiocytic lymphoma U937 cells	Fibrosis regression	(47)
PSTPIP2	Innate immune regulator involved in actin based cytoskeletal functions	Overexpression	STAT1 inhibition and STAT6 activation, in RAW264.7 cells	Attenuated fibrosis	(49)
RNF41	E3 ubiquitin ligase which promotes anti-inflammatory macrophage polarization	Macrophage-specific overexpression	Induction of IGF-I and HGF in macrophages	Fibrosis regression	(4)

Bold type indicates findings or targets supported by evidence from human cells, human samples, or patient studies.

Additional targets include immune receptors and signaling amplifiers such as interferon alpha/beta receptor subunit 1 (IFNAR-1) (59) and triggering receptor expressed on myeloid cells-1 (TREM-1) which is also upregulated in **human** cirrhotic livers (95), as well as ion channels such as voltage-gated potassium channel Kv1.3 (96). Collectively, these findings reinforce the concept that selective inhibition of pro-fibrotic macrophage programs can effectively promote a shift toward restorative phenotypes and attenuate liver fibrosis.

An elegant gene-delivery strategy was developed by Melgar-Lesmes et al. (97), who engineered graphene nanostars conjugated to a PAMAM-G5 dendrimer to selectively deliver an MMP-9-encoding plasmid under the control of the CD11b promoter to inflammatory, pro-fibrotic macrophages in cirrhotic livers. Although MMP-9 is typically upregulated during fibrogenesis, its targeted overexpression in macrophages induced a phenotypic switch toward a pro-regenerative restorative state both *in vitro* and *in vivo*. This approach selectively reduced collagen deposition in fibrotic regions enriched in inflammatory macrophages, while leaving HSC activation largely unaffected.

Conversely, several studies reported that genes downregulated during fibrogenesis exert antifibrotic effects when restored or overexpressed in the CCl_4_ model through macrophage-dependent mechanisms (**Table 1**). These include secreted factors such as interleukin-22 (IL-22), which correlates with a restorative macrophage phenotype in **human** samples (47); regulators of inflammasome activity such as proline-serine-threonine phosphatase-interacting protein 2 (PSTPIP2), predominantly expressed in macrophages (49); and enzymes such as the ring finger protein 41 (RNF41), which is also downregulated in macrophages from **human** cirrhotic livers (4).

### NAFLD/NASH (MASLD/MASH) models

Mouse *in vivo* models of non-alcoholic fatty liver disease (NAFLD; currently termed MASLD) and its progressive form non-alcoholic steatohepatitis (NASH; now MASH) -characterized by steatosis, inflammation, hepatocellular injury (ballooning), and frequently fibrosis- have been extensively used to investigate macrophage-driven fibrosis inhibition and/or resolution (**Table 2**).

**Table 2:** Genetic and pharmacological modulation inducing a resolutive phenotype in macrophages in MASLD/MASH mouse models.

Gene/pathway	Primary function/pathway	Type of modulation	Macrophage signaling pathways involved	Impact on fibrosis	Reference
Cathepsin B (CatB)	Lysosomal protease implicated in chronic diseases	Genetic knock-out	Non-determined	Attenuated fibrosis	(98)
CHIT1	A glycosidase, known as a pro-fibrotic macrophage marker	Pharmacological inactivation	Reduce glycolytic activity in naïve and LPS polarized BMDMs	Attenuated fibrosis	(26)
CCL2 (MCP-1)	Recruitment of CCR2^+^ proinflammatory and pro-fibrotic monocytes	Pharmacological inhibition and genetic knock-out	Non-determined	Attenuated fibrosis (strain-dependent)	(102, 115)
CCL3 (MIP-1α)	Pro-inflammatory chemokine secreted by macrophages	Myeloid-specific knock-out	NF-*κ*B, MAPK, JNK, ERK and eIF2α inhibition	Attenuated fibrosis	(103)
DPP-4 (CD26)	Cleaves many chemokines and peptide hormones	Pharmacological inhibition	NF-*κ*B, MAPK and ERK inhibition, *in vivo*. NF-*κ*B, JNK and c-JUN inhibition, in RAW264.7 cells	Attenuated fibrosis	(99, 100)
Ferroptosis	Iron-dependent cell death closely related to oxidative stress, inflammatory responses, and autophagy	Genetic or pharmacological inhibition	Non-determined	Attenuated fibrosis	(104)
FOXO1	Key transcription factor that acts as a substrate of Akt to mediate insulin effects	Myeloid-specific deletion	Release of STAT6 activity, in THP-1 **human** cells. Increase in STAT6, PPARδ, PPAR-γ and pro-resolutive markers expression levels in macrophages from **patients**	Attenuated fibrosis	(66)
Galectin-12 (LGALS12)	Lipid metabolism; inflammation	Genetic knock-out and knock-down	STAT6 activation and upregulation of SOCS3 expression levels, in THP-1 **human** cells	Attenuated fibrosis	(107)
HMGB1	Pro-inflammatory alarmin (DAMPs family member)	Macrophage-specific knock-down	Non-determined	Attenuated fibrosis	(108)
MD2 (TLR4 co-receptor)	A critical co-receptor of Toll-like receptor 4 (TLR4); innate immune signaling	Pharmacological inhibition	TLR2/4-MyD88, NF-kB and TBK1 inhibition, and AMPK activation, *in vivo*	Fibrosis regression	(37)
MLL4 (KMT2D)	Critical epigenetic regulator in the progression of NASH	Myeloid-specific deletion	NF-kB inhibition, in BMDMs	Near-complete protection from MASH	(14)
PARP1	Key enzyme involved in DNA repair processes and coactivator of NF-kB	Pharmacological inhibition	Attenuates the decrease in SIRT1 activation/expression, *in vivo*	Attenuated fibrosis	(101)
STING (TMEM173)	Signaling molecule eliciting type I interferon (IFN) immunity	Myeloid specific genetic disruption	JNK and NF-κB inhibition, in BMDMs	Attenuated fibrosis	(109)
XBP1	ER stress and unfolded protein response (UPR) transcription factor	Myeloid-specific knockout and pharmacological inhibition	STAT1 inhibition and STA6 activation, *in vivo*	Suppresses the development of steatohepatitis	(105, 106)
A1AT	A serine protease inhibitor involved in immune regulation	Gene restoration/supplementation	MAPK (Erk, JNK) and NF-κB (IkBα, p65) increased activation in primary KCs from A1T1^-/-^ mice	Attenuated fibrosis	(110)
METTL4	A methyltransferase controlling mRNA stability (RNA m6Am modification; splicing)	Overexpression	MyD88/NF-κB inhibition in THP-1 **human** cells	Attenuated fibrosis	(111)
Testosterone	Hormonal immunometabolic regulation	Supplementation	Non-determined	Attenuated fibrosis	(112)
CX3CR1	Chemokine receptor of CX3CL involved in monocyte-derived macrophages survival and polarization	Genetic knock-out	NF-κB and MAPK augmented activation, in peritoneal macrophages	Aggravated fibrosis	(114)
IL-33	Alarmin; Treg induction; M2 polarization	Cytokine administration	Upregulation of PPARα mRNA expression levels, *in vivo*	Improved metabolism but worsened fibrosis	(118)
BH4	A cofactor for nitric oxide synthases (NOS), which avoid generation of superoxide anions	Restoration	Non-determined	Attenuated fibrosis	(119)
MMP10	ECM remodeling	Genetic overexpression	STAT3 activation, *in vivo*. Increase in PPARγ expression levels, in KCs and RAW264.7 cells	Attenuated fibrosis	(57)
Nr4A1	Transcription factor that regulates monocytes differentiation	Induced upregulation	Non-determined	Attenuated fibrosis	(120)
NRF2	Master regulator of the cellular adaptive response to oxidative stress	Genetic overexpression in macrophages	Non-determined	Attenuated fibrosis	(121)
TIM-4	Mediate efferocytosis by liver resident macrophages	Tim-4^+^ macrophage restoration/enrichment	Efferocytosis pathway	Fibrosis attenuation and/or regression	(7)
VSIG4	Block pro-inflammatory macrophage activation. Reprogram mitochondrial pyruvate metabolism	Transplantation of VSIG4^+^ macrophages	NF-κB (Ikk-α and Ikβ-α) inhibition, *in vivo*	Attenuated fibrosis	(122)

Bold type indicates findings or targets supported by evidence from human cells, human samples, or patient studies.

In these models, multiple markers or pathways are upregulated during fibrogenesis, and their genetic deletion or pharmacological inhibition *in macrophages* has been shown to ameliorate liver fibrosis. These include proteases and enzymes, such as cathepsin B (CatB), whose serum levels are also elevated in cirrhotic **patients** (98); dipeptidyl peptidase-4 (DPP-4/CD26) (99, 100); poly[ADP-ribose] polymerase 1 (PARP1), which is also activated in **human** cirrhotic livers (101); and the glycoprotein chitinase-1 (CHIT1) (26).

Key chemokines were also targeted, including C-C motif chemokine ligand 2 (CCL2; MCP-1), a ligand for ***CCR2*** that is also upregulated in **human** cirrhotic livers (102), and CCL3 (MIP-1α) (103). In addition, metabolic pathways such as ferroptosis contribute to disease progression, and their modulation has been associated with fibrosis attenuation (104).

Several transcription factors and epigenetic regulators have been also analyze, including forkhead box O1 (FOXO1), which is also increased in **human** cirrhotic livers (66); mixed-lineage leukemia protein 4 (MLL4/KMT2D) (14); and X-box binding protein 1 (XBP1), which is also upregulated in MASH **patients** (105, 106). And also secreted factors such as FSTL1 (see above) (25) and galectin-12 (LGALS12) (107).

Finally, some studies analyzed the effect of inhibiting or deleting pattern recognition molecules and innate immune sensors including high mobility group box 1 (HMGB1) (108), myeloid differentiation factor 2 (MD2) (37), and stimulator of interferon genes (STING/TMEM173), which is also elevated in MASLD **patients**) (109).

Other genes expressed in hepatocytes -and *indirectly* affecting macrophage function- are downregulated during MASLD/MASH progression, and their restoration has been shown to improve disease outcomes (**Table 2**). These include alpha-1 antitrypsin (A1AT), a protective factor found also downregulated in cirrhotic **patients** (110). Notably, A1AT deficiency leads to upregulation of proteinase 3 (PR3), associated with increased recruitment of Mo-KCs, expressing this protease. PR3 cleaves IL-32γ, converting it into a potent activator of KCs. In the same study, pharmacological inhibition of PR3 ameliorated liver fibrosis in MASH. Other examples include the epigenetic regulator methyltransferase-like 4 (METTL4), which is also decreased in MAFLD **patients** (111), and testosterone, which may also exert protective effects (112).

The chemokine receptor CX3CR1, a key survival signal for hepatic monocyte-derived macrophages, is downregulated in liver samples from cirrhotic patients (113). However, Ni et al. reported that both CX3CL1 and its receptor CX3CR1 are upregulated in MASH (114), with CX3CR1 predominantly expressed by activated macrophages. Functionally, CX3CR1 deficiency aggravated disease severity, whereas CX3CL1 overexpression conferred protection. Similarly, Karlmark et al. (113) observed exacerbated fibrosis in CX3CR1^-/-^ mice in both CCl_4_ and BDL models, despite an overall reduction of CX3CR1 expression during fibrogenesis.

Interestingly, genetic deletion of CCL2 in CX3CR1^-^/^-^ mice ameliorated fibrosis by limiting macrophage infiltration and promoting restorative macrophage polarization, consistent with the antifibrotic effects of CCL2 inhibition (102). However, these effects were strain dependent, promoting reparative macrophage phenotypes in BalbC mice but not in C57Bl/6 animals (115), underscoring the importance of genetic background in shaping macrophage responses.

Additional complexity is illustrated by Chen et al. (116), who showed that macrophages engineered to express a *Toxoplasma gondii*-derived cytokine attenuate fibrosis through a sequential process involving early recruitment of Ly6C^hi^ macrophages followed by CX3CL1-dependent polarization toward a reparative Ly6C^lo^ phenotype. Similarly, Zhang et al. (117) demonstrated that the TCM formula FZHY modulates macrophage chemokine signaling by enhancing CX3CL1 while suppressing CCL2, thereby promoting a restorative phenotype.

However, these findings also underscore the need for caution when therapeutically targeting macrophage polarization. As shown by Weng et al. (65), IL-4Rα deficiency -despite its role in monocyte-to-resolutive macrophage differentiation- can paradoxically exacerbate fibrosis when present during active fibrogenesis, emphasizing the stage- and context-dependent nature of macrophage reprogramming.

Finally, Gao et al. (118) reported that interleukin-33 (IL-33) is upregulated in liver fibrosis. Although IL-33 promotes restorative macrophage polarization, expansion of tolerogenic ST2^+^ Tregs, and epithelial regeneration, its administration improved metabolic parameters but paradoxically worsened fibrosis, further illustrating the complexity of immune modulation in chronic liver disease.

Several additional genes are downregulated during MASLD/MASH progression, and their restoration has been shown to improve disease outcomes (**Table 2**). These include antioxidant and metabolic cofactor such as tetrahydrobiopterin (BH4) (119); matrix remodeling enzymes such as matrix metalloproteinase-10 (MMP10; stromelysin-2) (57); transcription factors including nuclear receptor subfamily 4 group A member 1 (Nr4A1) (120) and nuclear factor erythroid 2–related Factor 2 (NRF2; NFE2L2) (121); immune receptors such as T-cell immunoglobulin and mucin domain–containing molecule-4 (TIM-4), which mediates efferocytosis (7), and V-Set and immunoglobulin domain–containing 4 (VSIG4), a marker of KCs that is also decreased in MAFLD **patients** (122); and ubiquitin-related proteins such as tripartite motif-containing protein 38 (TRIM38) (86).

### Additional *in vivo* models

Other *in vivo* models -including alcoholic liver disease, bile-duct ligation, biliary senescence, thioacetamide injury, doxorubicin toxicity, and type-2 *diabetes mellitus* (T2DM)- have identified additional regulators of macrophage-driven fibrosis regression (**Table 3**).

**Table 3:** Macrophage-associated molecular targets modulating fibrosis in diverse in vivo disease models of other etiologies.

Gene/pathway	Disease model(s)	Primary function/pathway	Type of modulation	Macrophage signaling pathways involved	Impact on fibrosis	Reference
C/EBPβ	Alcoholic liver disease; toxic liver injury	Transcriptional regulation of inflammation and stress responses	Hepatocyte-specific knockout	Non-determined	Attenuated fibrosis	(79)
miR-155	Alcoholic liver disease; inflammatory injury	An alcohol-induced regulator of increased KC activation and TNFα production in macrophages	Genetic knock-out	Increased C/EBPβ and STAT3 expression levels, in KCs. TLR-4 pathway inhibition	Attenuated fibrosis	(123)
HNF4A	CCl_4_ and cholestatic liver injury	Hepatocyte differentiation and xenobiotic metabolism	Gene restoration/overexpression	Non-determined	Attenuated fibrosis	(124)
IGF-I	Thioacetamide and BDL	Growth factor; tissue repair	Gene or protein restitution	Non-determined	Fibrosis inhibition and regression	(125)
PPARα	Monogenic disease	Nuclear hormone receptor involved in mitochondrial fatty acid β-oxidation	Activation	Non-determined	Fibrosis inhibition and/or regression	(30)
TGF-β1	Bile-duct ligation	Canonical profibrotic cytokine	Cytokine administration	Decreased NF-κB expression levels, in hepatocytes and KCs	Attenuated fibrosis markers	(126)
HO-1	Zucker diabetic fatty rats	A microsomal antioxidant and cytoprotective enzyme	Induction/overexpression	Non-determined	Attenuated fibrosis	(128)

Among factors upregulated during fibrogenesis, whose deletion or inhibition promotes fibrosis attenuation or regression, are transcriptional and post-transcriptional regulators such as CCAAT/enhancer binding protein β (C/EBPβ), which is targeted *in hepatocytes* but influences macrophage phenotype through HDL remodeling (79), and miR-155, a potent driver of pro-fibrotic macrophage polarization (123).

Conversely, restoration of factors downregulated during disease progression has also been shown to exert antifibrotic effects. These include hepatocyte-derived protective regulators, such as hepatocyte nuclear factor 4 alpha (HNF4A), which modulates macrophage phenotype via paraoxonase secretion (124), and peroxisome proliferator–activated receptor-α (PPARα) (30), as well as endocrine/growth factors such as IGF-I (125). Collectively these approaches promote proresolutive macrophage polarization and contribute to fibrosis inhibition or resolution.

Interestingly, although transforming growth factor-β1 (TGF-β1) is a central driver of fibrogenesis, its administration reduced fibrosis in a BDL model by promoting macrophage polarization toward a restorative phenotype (126). In contrast, TGF-β1 inhibition attenuated liver fibrosis in a biliary senescence model (127), underscoring the strong context dependence of its effects.

Finally, induction of heme-oxygenase (HO-1), a cytoprotective antioxidant enzyme, exerts antifibrotic effects, further supporting the role of redox regulation in macrophage reprogramming and fibrosis resolution (128).

## Potential risks of macrophage reprogramming

While promoting pro-resolutive macrophage phenotypes represents a promising therapeutic strategy, potential risks must be carefully considered. The functional consequences of macrophage polarization in liver fibrosis are highly context dependent. Although alternatively activated macrophages exert predominantly antifibrotic and pro-resolutive effects in metabolic and toxic injury models, their roles differ substantially in chronic viral hepatitis or parasitic infections. In these settings, excessive anti-inflammatory polarization may impair pathogen clearance and perpetuate chronic inflammation, thereby reshaping macrophage function (129–135).

For example, in alveolar echinococcosis, inhibition of Notch signaling aggravates liver fibrosis (136), and macrophage depletion using clodronate-loaded liposomes prior to *Echinococcus multilocularis* infection impairs parasite clearance while increasing parasite burden (137). These findings highlight a critical role for pro-fibrotic macrophages during the early phase of infection, where they contribute to pathogen elimination. Conversely, a shift toward restorative macrophages may favor immune tolerance, parasite persistence, and fibrosis progression (138, 139).

Notably, divergent outcomes have been reported depending on the infectious context. In *Schistosoma japonicum* infection, Ren et al. (140) showed that treatment with IL-37 ameliorates liver pathology and is associated with enhanced restorative macrophage polarization *in vivo*. Mechanistically, IL-37 induces a pro-resolutive phenotype through AMPK phosphorylation *in vitro*, suggesting that metabolic reprogramming of macrophages can still confer benefit in selected infection-associated fibrosis settings.

Collectively, these observations underscore that macrophage-targeted therapeutic strategies must account for disease etiology and inflammatory context. Approaches aimed at promoting restorative macrophage phenotypes may be advantageous in sterile or metabolic liver injury but could be detrimental when effective pathogen clearance depends on sustained pro-inflammatory macrophage activity.

Moreover, macrophages with pro-resolutive or immunoregulatory profiles may contribute to tumor progression by supporting immune evasion, angiogenesis, and tissue remodeling. In **humans**, tumor-associated macrophages (TAMs) exhibit both pro- or anti-tumorigenic functions depending on the microenvironment (141, 142).

These context-dependent effects highlight the need for precise temporal and disease-specific modulation of macrophage responses.

## Other strategies to promote liver fibrosis amelioration

Several small molecules and bioactive compounds have been shown to ameliorate liver fibrosis in murine models by modulating macrophage polarization and function (**Table 4**). In some cases, antifibrotic effects are achieved primarily through interference with macrophage polarization toward both pro-fibrotic or pro-resolutive phenotypes, without directly targeting HSC activation. Examples include glucosamine (143) and chitooligosaccharides, low–molecular-weight oligomers derived from chitosan hydrolysis (144). In contrast, other compounds, such as the TCM formula Si-Wu-Tang, exert dual effects by also directly promoting HSCs deactivation or apoptosis (145).

**Table 4:** Therapeutic strategies promoting restorative macrophage phenotypes in experimental liver fibrosis.

Intervention/compound	Experimental model	Main target/pathways	Effect on macrophages	Antifibrotic mechanism
Glucosamine	CCl_4_ mouse	Immunomodulation (unspecified)	Inhibits their polarization	Reduced inflammatory macrophage activity
Chitooligosaccharides	CCl_4_ mouse	JAK2/STAT1 and JAK1/STAT6 inhibition in KCs	Inhibits their polarization	Attenuation of macrophage-driven fibrosis
Si-Wu-Tang (TCM)	CCl_4_ mouse	Multitarget (immune + HSC apoptosis)	Inhibits their polarization	HSC deactivation and apoptosis
Biejia Ruangan Tablet (BJRG; TCM)	CCl_4_ and BDL mouse	PDGF-AA/PI3K/AKT inhibition	Restorative phenotype polarization	Disruption of macrophage–HSC crosstalk
1, 25(OH)_2_Vitamin D_3_	MASH rat	PPARγ activation and NF-kB inhibition	Restorative phenotype polarization	Reduced inflammation and fibrosis
IFC-305 (adenosine derivative)	CCl_4_ rat	Arginine metabolism	Restorative phenotype polarization	Shift toward restorative macrophages
Myricetin	MASH mouse	NF-kB and STAT3 inhibition in LPS-stimulated RAW264.7 macrophages	Inhibits pro-fibrotic polarization	Reduced inflammatory signaling
Celastrol	MASLD mouse	Inhibit Warburg effects through suppressing AKT-, mTOR- and HIF-1α-mediated signaling pathways	Promotes oxidative phosphorylation-driven restorative phenotype	Metabolic reprogramming of macrophages
Lithocholic acid	CCl_4_ mouse	MAPK and NF-kB inhibition, *in vivo*. AKT and ACLY activation, in KCs	Restorative phenotype polarization (microbiota-dependent)	Reduced glycolysis, enhanced oxidative phosphorylation
Nobiletin	MASH mouse	RORα–KLF4 axis, in RAW264.7 cells. No involvement of AMPK signaling	Restorative phenotype polarization	Transcriptional reprogramming of macrophages
Honokiol	MASH mouse	PPARγ activation, in RAW264.7 cells	Restorative phenotype polarization	Redox control and immune modulation
Carotenoids	MASH mouse	Strong inhibition of MAPK, NF-kB and JNK in KCs	Restorative phenotype polarization	Reduced oxidative inflammation
Omega-3 lipid mediators	Various models	RORα and Nrf2 activation and NF-kB inhibition	Restorative phenotype polarization	Resolution of inflammation
Ketone ester feeding	MASLD mouse	Likely through NF-kB inhibition	Restorative phenotype polarization	Immunometabolic shift
Dasatinib	MASH mouse	Tyrosine kinase inhibition	Restorative phenotype polarization	Attenuated lipogenesis and macrophage reprogramming
Imatinib	CCl_4_ rat	Enhanced STAT6 expression	Ly6C^low^, Arg-1^+^ macrophages	Macrophage reprogramming
TCM formulations (BJXZ, GGCLT, JTCD, QZD, YZH)	MASH and CCl_4_ mouse, and CCl_4_ rats	TLR-4/MyD88/NF-kB inhibition, SIRT1 activation, and increased in Nrf2, HO-1, SIRT1, PGC-1α and OPA1 expression levels in RAW264.7 cells or *in vivo*. Inhibition of Notch signaling in THP-1 **human** cells. Inhibition of PI3K/AKT/mTOR, in rats *in vivo*	Restorative phenotype polarization	Immunometabolic restoration
PPARγ agonist GW1929 (nanotherapy)	CCl_4_ mouse	PPARγ activation in macrophages, *in vivo*	Restorative phenotype polarization	Selective macrophage reprogramming
MSCs/hAECs	Multiple murine models	Paracrine immunomodulation	Restorative phenotype polarization	Immune resolution, HSC inactivation
MSC-derived extracellular vesicles	CCl_4_ mouse	NF-kB inhibition, in THP-1 **human** cells	Restorative phenotype polarization	Immune reprogramming
Apoptotic MSC-derived EVs	CCl_4_ mouse	Efferocytosis pathways	Restorative phenotype polarization	Amplified regenerative signaling
MSC-derived TSG-6	CCl_4_ mouse	AKT, NF-kB and STAT1 inhibition, and STAT3 activation, in BMDM	Macrophage reprogramming	Immune reprogramming

Bold type indicates findings or targets supported by evidence from human cells, human samples, or patient studies.

Other compounds target macrophage-HSCs crosstalk. The patented TCM formulation Biejia Ruangan Tablet (BJRG) attenuates fibrosis by downregulating the PDGF-AA/PI3K/AKT signaling axis in restorative macrophages, thereby limiting profibrotic paracrine signaling toward HSCs (45). These findings highlight the central role of macrophage-derived signals in shaping the fibrotic niche.

Multiple interventions promote macrophage polarization toward a restorative phenotype, leading to improved fibrosis outcomes. Supplementation with 1, 25-dihydroxyvitamin D_3_ -reduced in **patients** with MASH- enhances restorative polarization in a choline-deficient diet–induced rat model via PPARα- and PPARγ-dependent pathways (73). Similarly, administration of IFC-305, an adenosine derivative, increases arginase activity while reducing iNOS expression in CCl_4_-treated rats, consistent with a shift toward a restorative phenotype (146).

A growing body of evidence supports the role of immunometabolic reprogramming in regulating macrophage polarization during fibrogenesis. Myricetin, a naturally occurring flavonol, suppresses TREM-1 and toll-like receptors 2/4 (TLR2/4)–MyD88 signaling, thereby attenuating pro-fibrotic macrophage activation (52). Celastrol, a natural triterpenoid, directly inhibits PKM2, promoting a metabolic shift from glycolysis toward oxidative phosphorylation and favoring restorative polarization (23). Similarly, lithocholic acid, a natural agonist of the bile acid receptor TGR5/GPBAR1, reduces glycolysis and enhances oxidative phosphorylation in a gut microbiota–dependent manner, promoting restorative macrophage polarization in a chronic CCl_4_ model (147).

Other natural compounds act through immunometabolic and transcriptional mechanisms. Nobiletin, a polymethoxylated flavonoid, upregulates krüppel-like factor 4 (KLF4) via activation of through retinoic acid–related orphan receptor α (RORα), thereby promoting restorative polarization and attenuating fibrosis in a MASH model (80). Honokiol, a natural biphenolic compound, enhances antioxidant defenses, downregulates cytochrome P450 2E1 (CYP2E1), and activates PPARγ, a key regulator of restorative macrophage differentiation (71). Additional agents -including ***carotenoids*** (29, 148–150), ***omega-3 lipid*** mediators (151), ketone ester supplementation (152), and the ***tyrosine kinase inhibitor*** dasatinib (153)- have also been shown to induce restorative macrophage phenotypes in mouse. Notably, imatinib, a tyrosine kinase inhibitor, promotes regression of CCl_4_-induced fibrosis in rats by inducing Ly6C^low^ macrophages with elevated Arg-1 and STAT6 expression, exceeding the efficacy of mesenchymal stromal/stem cell (MSC) therapy (64).

TCM formulations derived from herbal or fungal sources have consistently demonstrated antifibrotic effects associated with restorative macrophage polarization and enhanced mitochondrial function. These include Biejiaxiaozheng (BJXZ) (13); Ger-Gen-Chyn-Lian-Tang (GGCLT) (154); Jiawei Taohe Chengqi Tang (JTCD) (50); Qizhuyanggan Decoction (QZD) (46); Wuling capsule (155); Xiezhuo Tiaozhi (XZTZ) (156); Yi-Qi-Jian-Pi (YQJPF) (157); and Yinzhihuang (YZH) (54).

Nanotechnology- and cell-based approaches further highlight the immunoregulatory role of macrophages in fibrosis resolution. Moreno-Lanceta et al. (70) demonstrated that dendrimer-graphene nanostars conjugated to the PPARγ agonist GW1929 selectively activate macrophage PPARγ signaling and markedly reduced fibrosis in a CCl_4_-induced mouse model. Similarly, multiple stem and progenitor cell therapies -including **human** amniotic epithelial cells (158) and MSCs (125, 159, 160)- exert antifibrotic effects largely through macrophage reprogramming. Exosomes derived from **human** embryonic stem cell spheroids promote restorative polarization by suppressing the miR-1184/DICER1 axis (161), while extracellular vesicles are key mediators of MSC-induced immunomodulation and tissue repair (162, 163). Notably, induction of MSC apoptosis prior to vesicle collection enhances regenerative efficacy, likely via efferocytosis-driven macrophage reprogramming (164), and improving MSC homing further amplifies therapeutic outcomes (165). Wang et al. (166) identified tumor necrosis factor–stimulated gene 6 (TSG-6) as a critical MSC-derived factor, as its loss abrogates antifibrotic effects *in vivo*.

Collectively, these studies underscore macrophage plasticity as a central determinant of fibrosis progression and resolution. However, in many cases it remains unclear whether therapeutic benefit arises predominantly from suppression of pro-fibrotic macrophage programs or from activation of pro-resolutive macrophage functions, such as collagen degradation, efferocytosis, and immune resolution. Dissecting these mechanisms will be essential for the rational design of macrophage-targeted therapies for chronic liver disease.

## Macrophage-based therapy

Macrophage-based therapy is emerging as a promising strategy for the treatment of liver fibrosis and is currently under active investigation for clinical translation. Given the marked ontogenetic and functional heterogeneity of macrophages, Ma et al. (167) sought to identify the most suitable macrophage subset for cytotherapy. BMDMs were left unpolarized or differentiated *in vitro* into pro-fibrotic or restorative phenotypes and intravenously administered to mice at different stages of liver fibrogenesis induced by CCl_4_ or BDL. Infusion of either unpolarized or pro-fibrotic BMDMs significantly attenuated liver fibrosis, with pro-fibrotic macrophages exerting the strongest therapeutic effect, whereas restorative macrophages showed minimal efficacy. The superior activity was attributed to the ability of pro-fibrotic BMDMs to remodel immune cell interactions within the hepatic microenvironment, ultimately promoting HSC clearance and fibrosis resolution.

Li et al. (168) reported similar findings in a model of hepatic fibrosis induced by cystic echinococcosis. Pro-fibrotic BMDM infusion was associated with a phenotypic shift of endogenous infiltrating monocytes toward pro-resolutive macrophage phenotype, which mediate antifibrotic effects (1), although the underlying mechanisms in parasitic fibrosis remain incompletely understood. Consistent with these observations, Li and He (169) demonstrated that adoptive transfer of *in vitro*-expanded KCs into mice significantly reduced liver fibrosis in chronic CCl_4_-treated mice.

Watanabe et al. (3) further showed that co-infusion of MSCs and induced BMDMs (generated by CSF-1 stimulation) markedly enhanced regression of advanced fibrosis in a CCl_4_-model. This effect was associated with recruitment of endogenous macrophages and neutrophils, with the recruited macrophages displaying a pro-resolutive phenotype. Notably, the combined therapy produced a synergistic effect compared with either treatment alone, and BMDMs alone were more effective than MSCs alone.

Although most monocytes and macrophages infiltrating the liver during fibrogenesis originate from the bone marrow, additional populations derive from extrahepatic reservoirs such as the spleen or the peritoneal cavity, where they exhibit distinct functional properties (170). The spleen serves as a major monocyte reservoir that mobilizes in response to tissue injury (171), while peritoneal macrophages display high migratory capacity and can populate visceral organs, including the liver (172, 173).

Given this complexity, the development of macrophage-based therapies with stable and well-defined phenotypes is critical. Chen et al. (116) engineered macrophages to stably express migration inhibitory factor (MIF) derived from *Toxoplasma gondii*. These cells exhibited enhanced chemotaxis, reduced inflammatory responses, and superior antifibrotic efficacy compared with lipopolysaccharide (LPS)/IFN-γ–stimulated macrophages. Their effects were mediated, at least in part, through increased CCL2 expression via activation of the ERK/HMGB1/NF-κB signaling axis, promoting recruitment of endogenous macrophages to the fibrotic liver.

Junior et al. (174) generated MER tyrosine kinase (MERTK)^+/hi^ M2c macrophages by treating BMDMs with baicalin and evaluated their therapeutic potential in a mouse MASLD model. These macrophages, characterized by enhanced efferocytic capacity (175), improved multiple disease parameters following infusion, including fibrosis. Treatment was associated with increased hepatic HDL secretion, reduced circulating CD4^+^ helper and CD8^+^ cytotoxic T-cell populations, and establishment of a pro-resolving immune microenvironment, as confirmed by transcriptomic profiling.

Finally, Dai et al. (176) engineered BMDMs to express a chimeric antigen receptor (CAR) targeting urokinase-type plasminogen activator receptor (uPAR), enabling selective targeting of activated HSCs in both CCl_4_- and MASH-induced fibrosis models. CAR-macrophage infusion significantly enhanced fibrosis regression compared with control cells, with increased fibrolytic activity. In addition, these cells promoted antigen presentation and T- cell activation, contributing to antifibrotic immune responses that were partially required for their therapeutic efficacy.

## Strategies that may lead to improved therapeutic outcomes

Fiore et al. (125) reported that, within hours of intravenous administration of MSCs genetically engineered to express IGF-I, liver macrophages rapidly adopted a pro-resolving phenotype in mice subjected to thioacetamide-induced injury (2 or 8 weeks). This early shift was characterized by increased expression of IL-10, arginase I, IGF-I, and HGF, along with suppression of pro-inflammatory cytokines, and was accompanied by a transient increase in hepatocyte proliferation. In contrast, HSCs inactivation occurred at later time points, as evidence by reduced α-smooth muscle actin expression beginning approximately 3 days after treatment. In addition, restorative macrophages were shown to protect hepatocytes from apoptosis (177).

Collectively, these findings suggest that the therapeutic benefits of many antifibrotic interventions may initially arise from macrophage reprogramming toward a pro-resolutive phenotype, which subsequently drives secondary effects, including enhanced hepatocyte survival and proliferation, as well as deactivation or elimination of activated HSCs. However, most studies evaluating antifibrotic strategies have not systematically addressed the temporal dynamics of cellular responses or directly compared effects across distinct hepatic cell populations. This limitation hampers a clear understanding of whether fibrosis inhibition or resolution is governed by a dominant mechanism or by multiple coordinated processes acting in parallel.

Effective antifibrotic strategies are therefore likely to require the simultaneous modulation of multiple hepatic cell types that cooperatively contribute to disease progression. In line with this concept, several pharmacological and biologically active compounds exert beneficial effects by acting in parallel on hepatocytes, macrophages, and HSCs, while restoring metabolic and inflammatory homeostasis.

Quercetin, a naturally occurring ***flavonoid*,** attenuates hepatic inflammation, oxidative stress, and fibrotic deposition in murine models by directly targeting HSCs and promoting macrophage polarization toward a restorative phenotype (62, 178). Similarly, soybean-derived polyenylphosphatidylcholine (PPC) exerts broad hepatoprotective effects by restoring membrane phosphatidylcholine content, improving organelle function, suppressing sterol regulatory element–binding protein-1–driven lipogenesis, and limiting CYP2E1-associated toxicity (179). Beyond hepatocytes, PPC modulates KCs and macrophage responses, restrains HSC activation, supports liver regeneration, and influences gut–liver axis signaling.

Qi et al. (68) demonstrated that Sappanone A, a flavonoid, ameliorates CCl_4_-induced liver fibrosis, at least in part through ***PPARγ***-dependent restorative macrophage polarization and protection against oxidative hepatocyte injury, although these findings rely on cell line-based systems. Similarly, Shao et al. (147) showed that lithocholic acid, a secondary ***bile acid***, promotes inactivation of both HSCs and macrophages by inhibiting glycolysis and enhancing oxidative phosphorylation, thereby favoring restorative polarization; with gut microbiota playing a critical role *in vivo*.

Pirfenidone, a broad-spectrum antifibrotic agent, promotes restorative macrophage polarization, reduces HSC activation, and partially reverses established fibrosis in MASH models (180). Combined targeting of Notch and CXCR4 pathways further demonstrated synergistic antifibrotic effects through coordinated regulation of macrophage phenotype and fibrogenic signaling in a CCl_4_-induced mouse model (181).

Autophagy-centered interventions further emphasize the importance of immunometabolic regulation. Wang et al. (182) showed that overexpression of FAM134B, a receptor mediating endoplasmic reticulum (ER) autophagy, alleviates ER stress, suppresses inflammatory cytokine production, and limits activation of both macrophages and HSCs in alcoholic liver disease models.

The BJXZ pills, a ***TCM*** herbal formulation, exert multifaceted antifibrotic effects. They have been shown to alleviate oxidative stress in macrophages by inhibiting NF-κB, activating NRF2 and HO-1 pathways, restoring mitochondrial membrane potential, and enhancing ATP production, while simultaneously promoting HSCs deactivation and apoptosis (13).

Paeoniflorin, a bioactive monoterpene glycoside derived from *Paeonia* species, similarly exerts antifibrotic effects in CCl_4_-induced liver fibrosis in rats by promoting restorative macrophage polarization and HSC deactivation (15). Notably, both paeoniflorin and Sappanone A -compounds derived from plants used in TCM- upregulate SIRT1 and PGC-1α and induce AMPK phosphorylation, thereby supporting mitochondrial biogenesis and metabolic fitness (183, 184), whereas pro-inflammatory stimuli such as LPS-driven polarization suppress SIRT1 expression (185). In this context, SIRT6 emerges as a particularly promising therapeutic target, given its ability to regulate mitochondrial function, restrain NF-κB and NLRP3 signaling, support DNA repair and cell survival, prevent fibrogenesis, and promote pro-resolutive macrophage polarization (61).

Atriol, a ligand of the mitochondrial translocator protein (TSPO), restores mitochondrial function in hepatocytes from MASH rats, suppresses pro-inflammatory chemokine expression in macrophages, and reduces HSC activation through inhibition of NF-κB signaling (186).

We have shown that IMT504, an immunomodulatory oligodeoxynucleotide, exerts potent antifibrotic and pro-regenerative effects by simultaneously targeting all major cellular players in liver fibrosis (2). IMT504 reprograms inflammatory macrophages toward a restorative phenotype, induces HGF and IGF-I expression in hepatocytes, and promotes HSC deactivation. In addition, it enhances the proliferation and recruitment of GLAST^+^ Wnt1^+^ bone marrow stromal progenitors, further contributing to tissue repair.

At the molecular level, Rodríguez et al. (187) demonstrated in **human** B cells that IMT504 induces transcriptional programs associated with mitochondrial function, proteostasis, and antioxidant defense, governed by NRF1, NRF2, SIRT1, and PGC-1α. Notably, IMT504 was shown to bind ATP-citrate lyase (ACLY), a key enzyme in acetyl-CoA production and a negative regulator of AMPK, thereby potentially relieving ACLY-mediated inhibition of AMPK. This mechanism may provide a unifying explanation for its broad therapeutic effects (33, 34, 188).

Consistent with this concept, combined treatment with bempedoic acid (an ACLY inhibitor with antifibrotic activity) (189, 190), and the GLP-1RA liraglutide resulted in additive improvements in MASH and liver fibrosis in murine models (191). Mechanistically, ACLY inhibition limits lipid substrate availability (192), whereas GLP-1RA signaling modulates systemic metabolic and inflammatory pathways (193). Supporting this, ACLY expression is upregulated in macrophages during fibrogenesis (194), and its inhibition promotes a shift toward a restorative phenotype (195).

Overall, these findings indicate the therapeutic strategies incorporating mechanistic redundancy -simultaneously targeting macrophage immunometabolism, hepatocyte resilience, and HSC deactivation- are more likely to achieve more robust and durable antifibrotic effects than approaches focused on a single pathway or cell type.

## Translational and clinical evidence

The translational relevance of previous findings critically depends on whether macrophage subpopulations are evolutionarily conserved across the species, which appears to be the case. Single-cell RNA-seq studies conducted by independent groups have identified, in human MASLD, macrophage subsets largely analogous to those previously described in mouse (196–198). These include resident KCs (VSIG4^+^/CD163^+^/MARCO^+^/TIMD4^+^), Mo-KCs (VSIG4^+^/CD163^+^/MARCO^-^/TIMD4^-^), LAMs (VSIG4^-^/TREM2^+^/CD9^+^) and C-LAMs (VSIG4^-^/CX3CR1^+^/CCR2^+^). A minor population of IGSF21^+^ cells, corresponding to moKC precursors, has also been identified in both human and murine livers (196).

In MASLD and cirrhotic livers, monocyte, monocyte-derived macrophages and LAM precursors are significantly expanded, with LAMs accounting for a substantial fraction of the monocyte-derived macrophages (9, 196, 197). Spatially, LAMs preferentially localize to pericentral zones enriched in steatotic hepatocytes (196). Notably, TREM2^+^/CD9^+^ macrophages have been shown to exhibit a pro-fibrogenic phenotype and to accumulate within collagen-rich fibrotic niches in cirrhotic livers, leading to their designation as scar associated macrophages (SAMs) (198). In addition to TREM2 and CD9, SAM subsets express IL1β, SPP1, LGALS3, CCR2 and TNFSF12 (198), and appear to partially overlap with C-LAM populations. This phenotype likely represents a transitional state, predominantly derived from recruited monocytes, with the potential to differentiate into either LAMs or Mo-KCs (9). In both mice and humans, C-LAMs have been shown to organize into multicellular aggregates termed hepatic crown-like structures (hCLS), which are associated with the progression from simple steatosis to MASH (199).

Collectively, these findings support the concept that human macrophage populations recapitulate, at least to a certain extent, key features of their murine counterparts, suggesting that therapeutic strategies targeting macrophage subsets in preclinical models may have translational applicability in human liver disease. Consistent with this notion, numerous studies employing gene-targeting approaches have demonstrated that key macrophage-associated markers are similarly upregulated or downregulated during fibrogenesis in both mice and humans, reinforcing the evolutionary conservation of these pathways. Moreover, in many instances where gene-targeting strategies successfully promoted a restorative macrophage phenotype in murine models, their application to human cells or tissues yielded comparable outcomes, involving overlapping of main signaling pathways, as discussed in a previous section (see also **Tables 1**, **2**).

Results from clinical trials that likely engage restorative macrophage activity -based on insights derived from murine models of liver fibrosis discussed above- have already been reported. The main molecules and signaling pathways known to induce a restorative macrophage phenotype, and that have been evaluated in clinical trials, are highlighted in **bold** and *italics*. A summary of the analysis herein made of reported clinical trials targeting liver fibrosis, with involvement of restorative macrophages and grouped by therapeutic class, is shown in **Table 5**. Therapeutic strategies are organized by class and aligned with their primary mechanisms of action and clinical development stage. For each kind of treatment, the table highlights study design, patient population, key endpoints, and reported effects on liver fibrosis.

**Table 5:** Clinical trials targeting liver fibrosis grouped by therapeutic class.

Therapeutic class	Intervention (year)	Phase/design	Population	Key endpoints	Fibrosis outcome
**Bile acids**	UDCA (1994–1996)	Clinical studies	CF-related liver disease; early PBC	Liver function tests	Not assessed; modest functional improvement
**PPARγ agonists**	Pioglitazone (2006, Belfort)	RCT	MASH + T2DM/IGT	Histology	No significant improvement
	Pioglitazone (2008, Aithal)	RCT	Non-diabetic MASH	Histology	Significant improvement
	Rosiglitazone (2011, Torres)	Clinical study	MASH	Histology	Improvement comparable to Pioglitazone
**Natural compounds/nutraceuticals**	QYHTTLD (2008, Zhang)	Controlled study	MASH	Serum biomarkers	Not assessed
	Curcumin (multiple studies; incl. 2019, Saadati)	RCTs	MASLD/MASH	Metabolic and inflammatory markers. NF-kB activity in PBMCs and histology	Modest improvement
	Meriva^®^ curcumin (2025, Musso)	Double-blind RCT	MASH	Histology	Significant improvement
	Anthocyanins (2015, Zhang)	RCT	MASLD	Metabolic parameters	Not assessed
	Silymarin (2017, Kheong)	Double-blind RCT	MASH	Histology, liver stiffness	Significant improvement
	Fucoidan + Fucoxanthin (2021 Shih)	Clinical study	MASLD	Liver stiffness, metabolic markers	Improved fibrosis markers
**Omega-3 fatty acids**	EPA (2008, Tanaka)	Clinical study	MASLD/MASH	Histology	Improvement in subset
	Ethyl-EPA (2014, Sanyal)	Phase 2b RCT	MASH	Histology	No benefit
	DHA + Vitamin D (2016, Della Corte)	Clinical study	Pediatric MASLD	MASLD activity score	No significant stage change
**Cell-based therapies**	Monocyte infusion (2025, Brennan)	Phase II	Cirrhosis	Liver function, cytokines	No significant improvement
	Bone marrow–derived cells (2011, Saito)	Clinical study	Alcoholic cirrhosis	Liver function, histology	Improvement observed
**Targeted antifibrotic/anti-inflammatory agents**	Selonsertib ± Simtuzumab (2018, Loomba)	Phase 2	MASH (F2–F3)	Histology. Fibrosis progression	Numerical improvement only
	Cenicriviroc (CENTAUR)	Phase 2b RCT	MASH	≥1-stage fibrosis improvement	Significant, not sustained
**SGLT2 inhibitors**	Ipragliflozin (2022, Takahashi)	Clinical study	MASH	Histology	Significant improvement
	SGLT2 inhibitors (meta-analysis; 2025, Ahmed)	Systematic review	MASLD + T2DM	Histology	Consistent improvement
	Empagliflozin vs pioglitazone (2023, Attaran)	RCT	MASLD + T2DM	Fibrosis, steatosis	Comparable fibrosis reduction
	Empagliflozin (2024, Hooshmand Gharabagh)	Prospective study	MASLD	Fibrosis stage, stiffness	Improvement observed
**GLP-1 receptor agonists/incretin-based therapies**	GLP-1RAs (meta-analysis; 2024, Fang)	RCTs	MASLD/MASH	Histology	No worsening; limited fibrosis effect
	Tirzepatide (SYNERGY-MASH; 2024, Loomba)	Phase 2 RCT	MASH (F2–F3)	Histology. Resolution of MASH without worsening of fibrosis at 52 weeks.	Significant improvement
	Semaglutide (ESSENCE; 2025, Sanyal)	Phase 3	MASH (F2–F3)	Histology. Resolution of steatohepatitis without worsening of liver fibrosis and reduction in liver fibrosis without worsening of steatohepatitis	Significant improvement
**Combination metabolic therapies**	Exenatide + Dapagliflozin (2020, Gastaldelli)	Clinical study (104 weeks)	T2DM + MASLD	Steatosis, fibrosis markers	Superior improvement vs monotherapy

Bold type indicates findings or targets supported by evidence from human cells, human samples, or patient studies.

In 1994 and 1996, two studies evaluated the administration of ursodeoxycholic acid (UDCA), a hydrophilic secondary ***bile acid***, in patients with cystic fibrosis–associated liver disease (200) and early-stage primary biliary cirrhosis (201), respectively, reporting modest improvements in liver function.

In 2006, Belfort et al. (202) reported a randomized controlled trial (RCT) evaluating pioglitazone, a potent ***PPARγ agonist***, in 55 patients with impaired glucose tolerance or T2DM and MASH. Participants received either pioglitazone (45 mg/day) plus a hypocaloric diet or placebo plus diet for six months. Significant improvements were observed in hepatic steatosis, hepatocellular ballooning, necrosis, and inflammation; however, fibrosis reduction did not reach statistical significance compared with placebo. Two years later, Aithal et al. (203) conducted a similar 12-month RCT in nondiabetic patients with MASH. Histological analysis revealed significant reductions in hepatocellular injury, Mallory–Denk bodies, and fibrosis compared with placebo. Despite these encouraging findings, a meta-analysis published in 2012 encompassing data from four clinical trials concluded that, although pioglitazone consistently improves metabolic parameters and hepatic inflammation, its efficacy in promoting fibrosis regression is limited and variable (204). Moreover, concerns regarding adverse effects, including weight gain and long-term safety, constrain its widespread use as a sustained antifibrotic therapy in MASH. Collectively, these limitations highlight the need for alternative or combination therapeutic strategies specifically aimed at durable fibrosis resolution.

Subsequently, Torres et al. (205) reported that 48 weeks of rosiglitazone therapy, another PPARγ agonist, produced histological improvements in patients with MASH comparable to those observed with rosiglitazone combined with either metformin or losartan, including similar effects on steatosis, inflammation, ballooning degeneration, and fibrosis.

In 2008, Zhang et al. (206) compared the ***TCM*** herbal formulation QuYuHuaTanTongLuo Decoction (QYHTTLD) with UDCA, used as a control treatment, in patients with MASH treated for six months. The experimental treatment significantly ameliorated different inflammatory, metabolic and liver injury serum parameters. However, liver histology was not assessed. Subsequently, Saadati et al. (207) showed that curcumin supplementation (1500 mg/day for 12 weeks), a component of TCM formulations, reduced hepatic fibrosis and improved metabolic parameters, although it was not superior to weight loss alone in reducing cardiovascular risk. Follow-up studies confirmed reductions in fibrosis and NF-κB activity but no significant effects on steatosis or serum liver enzymes compared with lifestyle modification (208). In 2025, Musso et al. (209) reported that Meriva^®^, a phospholipid-formulated curcumin, significantly improved liver histology and reduced fibrosis in patients with MASH in a double-blind trial.

At least three clinical studies have evaluated ***long-chain omega-3 polyunsaturated fatty acids*** in MASLD/MASH. Tanaka et al. (210) reported that 12-month administration of eicosapentaenoic acid (EPA; 2700 mg/day) reduced serum oxidative stress markers and improved histological features in most evaluated patients. However, a multicenter phase 2b RCT by Sanyal et al. (211) found no significant histological benefit of ethyl-EPA compared with placebo, despite reductions in serum triglycerides. In children with MASLD, Della Corte et al. (212) showed that combined docosahexaenoic acid and vitamin D supplementation improved metabolic parameters, MASLD activity score, HSC activation, and collagen content, without significantly altering fibrosis stage.

Early ***cell-based therapies*** primarily employed mesenchymal stem cells, hematopoietic stem cells, or heterogeneous cell populations containing pro-inflammatory and pro-fibrotic lineages (213). Although preclinical results were encouraging, randomized clinical trials in cirrhotic patients have shown inconsistent benefits. A phase II trial reported that infusion of bone marrow–derived monocytes did not significantly improve liver function, although circulating pro-inflammatory cytokines were reduced (213). Conversely, another study demonstrated that intravenous infusion of autologous bone marrow cells in alcoholic cirrhosis significantly improved serum albumin, total protein levels, prothrombin time, and fibrosis severity compared with controls (214).

In 2015, Zhang et al. (215) reported a double-blind randomized trial in which anthocyanin supplementation (320 mg/day), a ***flavonoid*,** significantly improved insulin resistance and liver injury markers in patients with MASLD. Two years later, Chan et al. (216) published a double blinded RCT in which silymarin -a complex mixture of flavonolignans and minor polyphenolic compounds- was administered to patients with MASH for 48 weeks. Silymarin treatment resulted in a significantly greater improvement in liver fibrosis compared with placebo, as assessed by histology and supported by reductions in liver stiffness measured by Fibroscan and other non-invasive fibrosis markers. Notably, these antifibrotic effects occurred in the absence of significant improvements in other key histological features of the disease. In addition, Shih et al. (217) demonstrated that combined fucoidan -a seaweed extract- plus fucoxanthin -a ***carotenoid*-** therapy reduced liver stiffness, steatosis, metabolic dysfunction, and inflammatory cytokines in patients with MASLD.

In 2018, Loomba et al. (218) published a phase 2, randomized, open-label study assessing the safety and efficacy of selonsertib, a ***tyrosine kinase inhibitor***, administered either alone or in combination with simtuzumab -a monoclonal antibody targeting lysyl oxidase-like 2 (LOXL2)- in patients with moderate to severe fibrosis associated with MASH. During the 24-week treatment period, patients receiving selonsertib demonstrated numerically higher rates of fibrosis improvement and reduced fibrosis progression compared with those treated with simtuzumab alone.

Also in 2018, Friedman et al. (219) administered cenicriviroc in patients with MASH. Cenicriviroc (CVC) is an oral, dual ***antagonist of CCR2*** and CCR5 that was developed to target monocyte recruitment and macrophage-driven inflammation and fibrogenesis in this disease. In the phase 2b CENTAUR trial, cenicriviroc treatment (150 mg/day) for 1 year resulted in a significantly higher proportion of patients achieving ≥1-stage improvement in liver fibrosis without worsening of MASH compared with placebo. However, the beneficial effects on fibrosis were not sustained after 2 years of treatment, and cenicriviroc failed to meet its primary endpoint in the subsequent phase 3 AURORA trial, leading to discontinuation of its clinical development for MASH (220).

In 2022, Takahashi et al. (221) showed that ipragliflozin, an ***SGLT2i***, significantly improved hepatic fibrosis and MASH resolution compared with standard care. In 2025, Ahmed et al. (222) published a systematic review evaluating the efficacy of SGLT2is in improving hepatic steatosis, liver enzymes, glycemic control, and liver histology in patients with T2DM and MASLD. Of the seven studies included, five assessed liver fibrosis, and all reported significant improvement, with a substantially higher proportion of treated patients (57-60%) achieving at least a one-stage reduction in fibrosis compared with controls (16%).

Fang et al. (223) assessed the efficacy of ***GLP-1RAs*** for the treatment of MASLD or MASH in a systematic review and meta-analysis of RCTs. Treatment effects in liver fibrosis were evaluated only in 2 RCTs, using liver biopsy. Overall, GLP-1RA therapy was associated with significant improvements in histologic resolution of MASH with no worsening of liver fibrosis. In the phase 2 SYNERGY-MASH trial, Loomba et al. (224) evaluated the efficacy and safety of tirzepatide, a dual GIP/GLP-1 receptor agonist, in patients with biopsy-confirmed MASH and moderate to severe fibrosis (F2–F3). Over 52 weeks, once-weekly tirzepatide significantly outperformed placebo in achieving the primary endpoint of MASH resolution without worsening of fibrosis. Key secondary analyses showed that tirzepatide also increased the proportion of patients achieving at least one-stage fibrosis improvement without MASH worsening (approximately 51–55% vs. 30% with placebo). Treatment was generally well tolerated, with predominantly mild to moderate gastrointestinal adverse events. These findings demonstrate robust histological benefits of tirzepatide on both steatohepatitis and fibrosis over one year, supporting its therapeutic potential in MASH and justifying larger, longer-term trials to confirm durability and long-term safety. In the ESSENCE phase 3 trial, semaglutide showed a significant antifibrotic effect in patients with MASH and stage F2–F3 fibrosis (225). At week 72, 36.8% of semaglutide-treated patients achieved at least a one-stage fibrosis improvement without worsening of steatohepatitis, compared with 22.4% in the placebo group. Semaglutide also increased the proportion of patients achieving the combined endpoint of steatohepatitis resolution and fibrosis regression, supporting a coordinated benefit on inflammatory injury and fibrogenesis. These findings provide strong clinical evidence that sustained GLP-1 receptor agonism can translate metabolic improvements into meaningful histologic fibrosis regression.

Interestingly, Luo et al. (226) published a systematic review and network meta-analysis (NMA) of RCTs comparing the effects of four pharmacological classes in patients with MASLD, including vitamin E (α-tocopherol and δ-tocotrienol), pioglitazone, GLP-1RAs, SGLT2is (dapagliflozin, empagliflozin, ipragliflozin, and tofogliflozin) and placebo. This NMA revealed that liraglutide, a GLP-1RA, ranked highest in reducing enhanced liver fibrosis (ELF) scores, whereas pioglitazone ranked highest in improving histological fibrosis stage.

Consistently, Attaran et al. (227) conducted a 24-week randomized, single-blind clinical trial comparing empagliflozin (10 mg/day), a highly selective SGLT2i, with pioglitazone (30 mg/day) in patients with T2DM and MASLD receiving metformin. Empagliflozin was more effective in improving hepatic steatosis, whereas both treatments showed comparable efficacy in reducing liver fibrosis. Consistently, Hooshmand Gharabagh et al. (228) reported in a six-month, open-label prospective study that empagliflozin or pioglitazone, each combined with metformin, led to comparable improvements in liver fibrosis stage and stiffness, although empagliflozin was associated with greater weight loss.

However, Ren et al. (229) performed a systematic review and NMA comparing 26 hypoglycemic agents for the treatment of MASLD patients across 37 studies. Empagliflozin was most effective in improving liver stiffness measurement and pioglitazone had limited benefits in different metabolic and liver fibrosis outcomes. And interestingly, in another systematic review and NMA comparing SGLT-2 inhibitors, GLP-1 RA, and DPP4 inhibitors, the two first classes may be preferred for T2D patients to reduce hepatocellular carcinoma incidence and other hepatic-related outcomes, including liver cirrhosis (230).

Finally, Gastaldelli et al. (231) reported that combined treatment with exenatide (GLP-1 analogue) and dapagliflozin (an SGLT2i) over 104 weeks produced greater improvements in hepatic steatosis and fibrosis markers than either agent alone in adults with T2DM inadequately controlled on metformin (104 weeks; n = 695). Combination of SGLT2 inhibitors and GLP-1RA may ameliorate liver fibrosis through convergent, indirect mechanisms that act upstream of HSC activation (232, 233). Both classes reduce hepatocyte lipotoxicity, oxidative and ER stress, and dampen inflammatory signaling (233, 234). They also improve the systemic metabolic milieu by reducing insulin resistance, adipose tissue inflammation, and free fatty acid flux to the liver (232, 233, 235). Despite these shared effects, their mechanisms diverge: GLP-1RA rely largely on incretin signaling and central appetite suppression, leading to substantial weight loss and strong resolution of steatohepatitis, with improvements in gut dysbiosis (223, 232, 233). In contrast, SGLT2 inhibitors exert weight-independent benefits through renal glycosuria–driven metabolic reprogramming, reduced insulin levels, increased fat oxidation, and systemic anti-inflammatory and hemodynamic effects (233, 235–237).

Among currently evaluated strategies, SGLT2 inhibitors—particularly when combined with GLP-1RA—represent the most robust and immediately translatable approach for liver fibrosis, supported by consistent antifibrotic effects, advanced clinical validation, and favorable safety profiles. In contrast, pioglitazone and macrophage-targeted agents demonstrate mechanistic promise but are limited by safety, variability, or lack of durability, underscoring the need for combination strategies aimed at sustained fibrosis resolution.

Overall, most early interventions, including bile acids, PPARγ agonists, and nutraceuticals, demonstrate variable or modest antifibrotic efficacy and often lack of sustained effects. In contrast, more recent metabolic therapies -particularly SGLT2 inhibitors and GLP-1 receptor agonists- show consistent and clinically meaningful improvements in fibrosis, likely mediated through systemic metabolic reprogramming and indirect modulation of hepatic inflammation and stellate cell activation. Targeted antifibrotic and macrophage-modulating agents exhibit mechanistic promise but have shown limited durability or translational success to date. Collectively, these findings support a shift toward combination and metabolism-centered strategies to achieve sustained fibrosis resolution.

**Table 6** summarizes emerging therapeutic approaches for liver fibrosis, organized according to their predominant stage of action along the disease continuum (inflammation, fibrogenesis, and resolution). For each therapeutic class, the principal mechanisms of action and their impact on macrophage biology are indicated, distinguishing between direct targeting of macrophage recruitment or activation and indirect modulation through systemic metabolic or inflammatory pathways. Fibrosis outcomes are categorized based on available clinical evidence, highlighting variability in efficacy and durability across interventions. Notably, while early-phase strategies primarily aim to limit inflammation and monocyte recruitment, and mid-stage interventions target fibrogenic signaling, the most consistent antifibrotic effects are observed with therapies that promote a restorative macrophage phenotype during the resolution phase, often via metabolic reprogramming. This framework underscores the importance of macrophage plasticity, disease stage, and etiological context in determining therapeutic efficacy and supports the rationale for combination strategies to achieve sustained fibrosis regression.

**Table 6:** Therapeutic strategies mapped to fibrosis stage and macrophage function.

Therapeutic class	Main target phase	Mechanism	Macrophage effect	Fibrosis outcome
Cell therapies	Variable	Immunomodulation	Direct	Inconsistent
Cenicriviroc	Inflammation	CCR2/CCR5 blockade	↓ Monocyte recruitment	Transient improvement
PPARγ agonists	Fibrogenesis	Metabolic + anti-inflammatory	Partial reprogramming	Variable
Nutraceuticals	Inflammation/Fibrogenesis	Anti-inflammatory	Mild modulation	Modest
Selonsertib	Fibrogenesis	Stress signaling inhibition	Mainly indirect	Limited
SGLT2 inhibitors	Resolution	Metabolic reprogramming	Mainly indirect restorative shift	Consistent improvement
GLP-1RAs	Resolution	Incretin + weight loss	Strong indirect effects	Significant improvement
Combination therapies	Resolution	Multi-pathway	Synergistic	Superior outcomes

Finally, in **Table 7** therapeutic strategies are ranked by translational potential integrating antifibrotic efficacy, clinical evidence, and feasibility. Metabolic therapies -particularly SGLT2 inhibitors/antagonists and GLP-1 receptor agonists- show the strongest profiles, whereas lower-ranking approaches are limited by inconsistent efficacy, lack of histological validation, or scalability challenges.

**Table 7:** Ranking of therapeutic strategies for liver fibrosis according to translational potential.

Rank	Therapeutic class	Evidence for fibrosis improvement	Highest level of clinical evidence	Key factors supporting translation	Main limitations	Overall translational potential
1	SGLT2 inhibitors + GLP-1 receptor agonists	Greater improvement in fibrosis markers than monotherapy in long-term trials	One large RCT plus convergent class-level evidence	Synergistic metabolic and anti-fibrotic mechanisms; durable effects; strong clinical feasibility	Limited histology-based fibrosis endpoints	High
2	SGLT2 inhibitors (empagliflozin, ipragliflozin)	Consistent ≥1-stage fibrosis improvement across RCTs and systematic reviews	RCTs + multiple systematic reviews/NMAs	Reproducible antifibrotic signal; favorable safety; weight-independent metabolic and anti-inflammatory effects; widely approved drugs	Fibrosis often assessed by non-invasive markers	High
3	GLP-1 receptor agonists	Improvement in fibrosis-related scores; high ranking in NMAs	RCTs + meta-analyses	Strong MASH resolution; substantial weight loss; good safety profile	Limited biopsy-proven fibrosis regression data	Moderate-high
4	Pioglitazone (PPARγ agonist)	Histological fibrosis regression in some RCTs	RCTs + meta-analysis	One of few agents with biopsy-proven fibrosis improvement	Variable efficacy; weight gain; long-term safety concerns	Moderate–high (mainly in combination)
5	Flavonoid-based therapies (curcumin formulations, silymarin)	Histology-supported fibrosis reduction in double-blind RCTs	Small double-blind RCTs	Direct antifibrotic effects; excellent tolerability	Short duration; limited replication and scale	Moderate
6	Cenicriviroc (CCR2/CCR5 antagonist)	≥1-stage fibrosis improvement at 1 year	Phase 2b RCT	Direct targeting of macrophage-driven fibrogenesis	Lack of durability; phase 3 failure	Low–moderate
7	Cell-based therapies	Variable fibrosis improvement in small studies	Phase II trials/observational studies	Strong mechanistic rationale; regenerative potential	Heterogeneity, scalability, regulatory complexity	Low
8	Omega-3 polyunsaturated fatty acids	Inconsistent or negative histological outcomes	Pilot studies + phase 2b RCT	Safe and well tolerated	Lack of consistent antifibrotic efficacy	Low
9	UDCA/TCM herbal formulations (non-histologic)	No direct fibrosis assessment or modest effects	Early clinical studies	Biologic plausibility	Lack of histology-based fibrosis data	Very low

Altogether, these findings support the notion that effective antifibrotic therapies converge on macrophage immunometabolism, where metabolic rewiring interfaces with redox-sensitive pathways such as NF-κB to regulate inflammatory tone and promote fibrosis resolution.

## Concluding remarks and future directions

**Figure 2:**
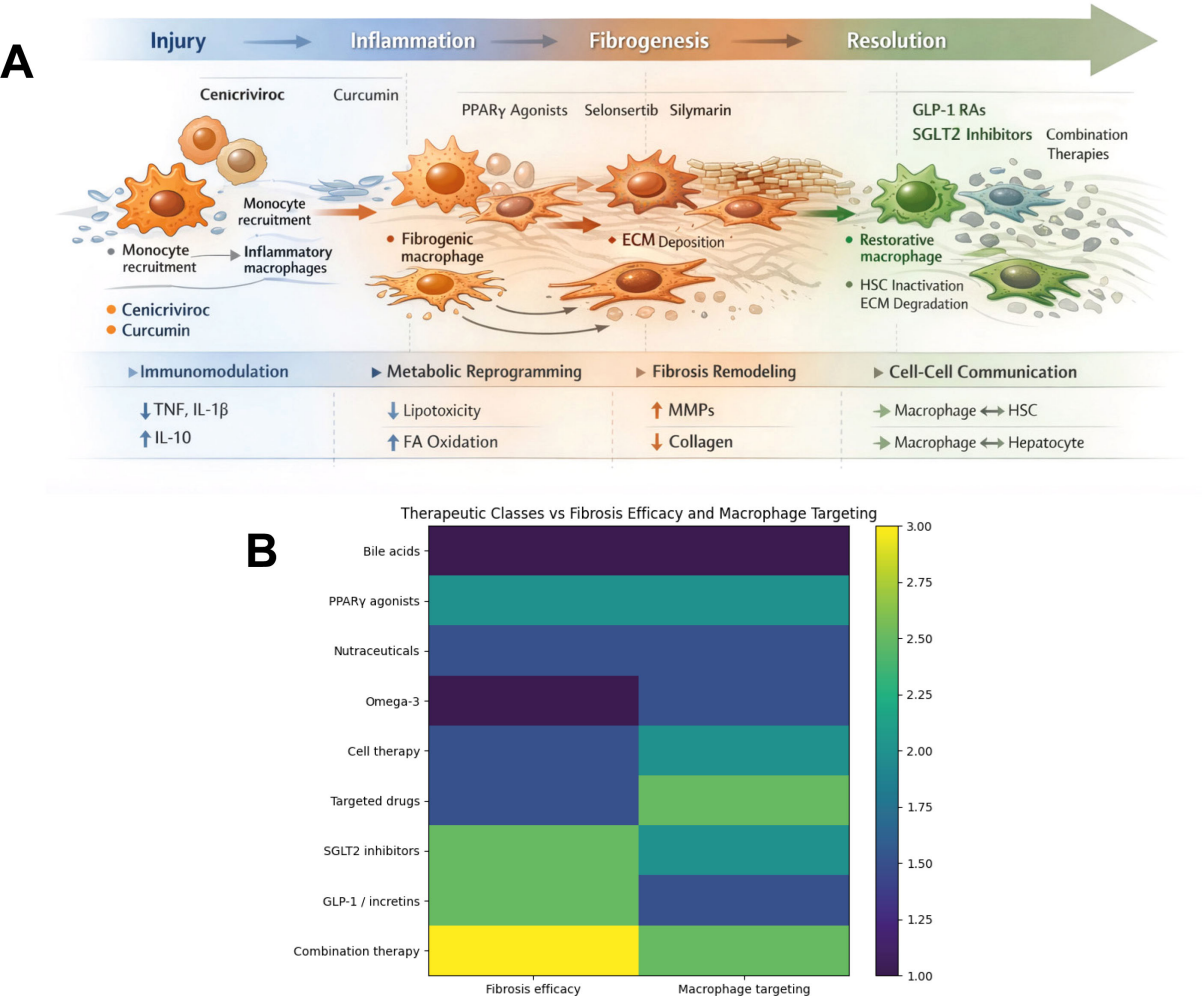
Macrophage-targeted and metabolism driven strategies across the continuum liver fibrosis. **(A)** This figure illustrates the progression of liver disease from injury to inflammation, fibrogenesis, and ultimately resolution, highlighting a dynamic spectrum of macrophage states from inflammatory to restorative phenotypes. It maps therapeutic strategies onto these phases, showing that early interventions (e.g., CCR2/CCR5 inhibition) target monocyte recruitment, whereas metabolic therapies such as GLP-1 receptor agonists and SGLT2 inhibitors act later to promote fibrosis resolution mainly indirectly. The lower panel integrates key mechanisms—including immunomodulation, metabolic reprogramming, extracellular matrix remodeling, and cell–cell communication—linking therapeutic effects to macrophage-driven regulation of hepatic stellate cells and tissue repair. **(B)** Graphical summary of antifibrotic therapies in clinical studies. Therapeutic classes are organized along the y-axis, while fibrosis efficacy and macrophage-targeting intensity are represented along the x-axis. Color gradients indicate relative magnitude of effect. Metabolic and combination therapies exhibit the highest antifibrotic efficacy, whereas targeted anti-inflammatory agents show stronger macrophage-directed activity but limited overall fibrosis improvement, highlighting the need for integrated immunometabolic strategies in MASH.

In summary, as depicted in **Figure 2**, liver fibrosis progression is a dynamic continuum spanning injury, inflammation, fibrogenesis, and resolution, highlighting the central role of macrophage plasticity. During early injury and inflammatory phases, circulating monocytes are recruited to the liver, giving rise to inflammatory macrophages that amplify tissue damage through pro-inflammatory cytokine production. In the fibrogenic phase, macrophages acquire pro-fibrotic functions that promote HSC activation and extracellular matrix (ECM) deposition. Transition toward resolution is characterized by the emergence of restorative macrophages, which facilitate ECM degradation, HSC inactivation or apoptosis, and tissue repair. Therapeutic strategies can be mapped onto these temporal phases. Cenicriviroc and anti-inflammatory compounds such as curcumin primarily target early monocyte recruitment and inflammatory signaling. PPARγ agonists, selonsertib, and silymarin act during fibrogenesis by modulating metabolic and stress-response pathways. In contrast, GLP-1 receptor agonists and SGLT2 inhibitors, alone or in combination, predominantly promote resolution through systemic metabolic reprogramming and indirect immunomodulatory effects that favor restorative macrophage phenotypes. Key underlying mechanisms are grouped into four functional modules: immunomodulation (reduction of TNF-α and IL-1β, increase in IL-10), metabolic reprogramming (reduced lipotoxicity and enhanced fatty acid oxidation), fibrosis remodeling (increased matrix metalloproteinase activity and decreased collagen deposition), and cell–cell communication among macrophages, HSCs, and hepatocytes. The impact of these pathways is further shaped by disease etiology, underscoring the context-dependent nature of macrophage-targeted therapies.

Although major advances have been made in elucidating the mechanisms by which macrophages acquire restorative phenotypes, much of the current knowledge derives from *in vitro* studies, frequently relying on the RAW264.7 mouse macrophage cell line. While useful for mechanistic exploration, this model has important limitations in the context of liver fibrosis. RAW264.7 cells do not faithfully recapitulate liver macrophage populations, as they lack the tissue-specific imprinting characteristic of KCs and monocyte-derived liver macrophages. Critical signals from the sinusoidal microenvironment, bile acids, and liver-derived cytokines that shape macrophage identity *in vivo* are absent, resulting in transcriptional and functional profiles that diverge from those of *bona fide* hepatic macrophages. Moreover, RAW264.7 cells originate from an Abelson leukemia virus-induced tumor in BALB/c mice (238) and exhibit constitutive activation of inflammatory and stress-related pathways, which may exaggerate or obscure antifibrotic responses. Finally, monoculture systems fail to recapitulate the complex cellular crosstalk among macrophages, HSCs, hepatocytes, and endothelial cells that governs fibrogenesis and its resolution. Thus, findings obtained in RAW264.7 cells should be interpreted with caution and ideally validated using primary hepatic macrophages, co-culture models, or *in vivo* models of fibrosis regression. Similar limitations apply to other macrophage-like cell lines, such as human THP-1, a monocytic leukemia-like cell line obtained from 1 year-old kid (239), or human histiocytic lymphoma U937 cells (240).

Accumulating evidence indicates that liver fibrosis is not merely the consequence of persistent metabolic injury, but rather the result of a dynamic immune–metabolic imbalance in which macrophages play a central role. Beyond the traditional M1/M2 paradigm, distinct macrophage subsets may exert either pro-fibrotic or restorative effects depending on their origin, metabolic programming, timing of activation, and interactions with other hepatic cell populations. Both experimental and clinical observations support the concept that effective fibrosis resolution requires macrophage reprogramming toward phenotypes that promote ECM degradation, hepatocyte survival and proliferation, and HSC deactivation or apoptosis.

Notably, a wide range of potential therapeutic strategies -including metabolic agents, nutraceuticals, immunomodulators, and cell-based therapies- appear to converge on shared molecular pathways regulating macrophage function. These include improved mitochondrial fitness, activation of AMPK-dependent signaling, attenuation of NF-κB and inflammasome-driven inflammation, and coordinated remodeling of lipid and glucose metabolism. Such convergence suggests that antifibrotic efficacy depends less on targeting a single pathway and more on restoring immune-metabolic homeostasis within the hepatic microenvironment. Importantly, these immunometabolic effects converge on redox-sensitive signaling nodes -including TRPM2 and NF-κB- which integrate metabolic stress with inflammatory responses and thereby critically regulate macrophage polarization and fibrosis outcomes. In this context, macrophages emerge as integrative hubs capable of translating metabolic and inflammatory cues into coordinated tissue repair responses.

As discussed throughout this work, the literature often focuses on individual signaling pathways in association with macrophage phenotypic changes, without fully addressing the broader network of antioxidative, metabolic, mitochondrial, and autophagy-related processes involved. These pathways interact extensively and may collectively influence fibrosis regression. For instance, PPARγ activation intersects with NF-κB, STAT1, and STAT6 signaling to regulate macrophage polarization (241). Similarly, AMPK activation inhibits non-canonical NF-κB signaling (IκB/p52), without affecting STAT3, ERK, JNK, or MAPK signaling pathways in KCs, as it was shown in a BDL rat model (242), whereas AMPK inhibition restores NF-κB signaling in LPS-treated FABP5-knockout macrophages (243). Together, these findings underscore the need for a more integrated understanding of the hierarchical network governing macrophages reprogramming toward restorative phenotypes. Such knowledge -particularly in the context of combination therapies- may facilitate the development of more effective antifibrotic strategies with higher translational potential.

The efficacy of therapeutic interventions in experimental models is highly dependent on disease context, with notable differences between toxic and metabolic fibrosis. In CCl_4_-induced injury, fibrosis is driven primarily by acute inflammation and rapid accumulation of monocyte-derived macrophages, making it highly responsive to single-pathway interventions. In contrast, MASH-associated fibrosis arises from a complex and chronic interplay between metabolic dysfunction, hepatocellular stress, and immune remodeling, characterized by greater macrophage heterogeneity and specialization. In this setting, therapeutic efficacy is more limited and typically requires combinatorial or systemic approaches capable of reshaping both immunological and metabolic circuits. These distinctions highlight the importance of model selection and underscore the need to prioritize strategies that target macrophage diversity and plasticity in metabolically driven disease.

Importantly, accumulating evidence indicates that macrophage subpopulations in murine and human livers share conserved features during fibrogenesis, and that several experimental strategies target common pathways to promote a shift from a pro-fibrotic to restorative phenotypes. Consistently, some of these strategies have demonstrated encouraging translational potential in clinical settings.

Despite robust preclinical evidence and increasingly positive clinical trial data, translation into routine clinic practice remains challenging. Clinical outcomes are heterogeneous, with variable response rates and durability. Moreover, most studies rely on static end-point analyses and fail to capture the temporal dynamics underlying successful tissue repair. Emerging evidence suggests that macrophage reprogramming may precede and facilitate hepatocyte regeneration and HSC inactivation, underscoring the need for time-resolved and cell-specific analyses. Future therapeutic strategies will likely require the coordinated modulation of multiple hepatic cell populations through pleiotropic agents and/or rational combination therapies to achieve synergistic antifibrotic effects.

At present, therapies combining robust antifibrotic efficacy, advanced clinical trial validation, and favorable safety profiles -particularly SGLT2 inhibitors alone or in combination with GLP-1 receptor agonists- represent the most promising and clinically translatable approaches. In contrast, macrophage-targeted therapies remain mechanistically compelling but are still limited by issues of durability and late-phase clinical validation.

In conclusion, a deeper understanding of macrophage-centered immune–metabolic networks -including mitochondrial function, autophagy, redox homeostasis, and epigenetic regulation-will be essential for the rational design of next-generation antifibrotic therapies. Future research should prioritize the identification of robust biomarkers reflecting macrophage phenotype, functional state, and spatial organization within the liver, enabling improved patient stratification and mechanism-guided combination therapies. Therapeutic strategies that actively harness macrophage plasticity to promote restorative programs -rather than merely suppressing inflammation- are more likely to achieve durable fibrosis regression and meaningful recovery of liver function. As our understanding of macrophage heterogeneity and context-dependent behavior continues to evolve, macrophage-centered, immune-metabolically informed interventions may ultimately redefine the treatment paradigm for chronic liver disease and cirrhosis.

## Glossary

ACLY: ATP-citrate lyase
AKT: protein kinase B; PKB
AMPK: AMP-activated protein kinase
Arg-1: arginase-1
AXL: AXL receptor tyrosine kinase
BMDMs: bone marrow–derived macrophages
BMSPs: bone marrow stromal progenitors
C/EBP: CCAAT enhancer binding protein
CatB: cathepsin B
CB1: cannabinoid receptor-1
CCL: C-C motif chemokine ligand
CCl_4_: carbon tetrachloride
CCR: C-C chemokine receptor
cGAS: cyclic GMP–AMP synthase
CHIT1: chitinase-1
COX2: cyclooxygenase-2
CSF-1: colony-stimulating factor-1
CXCR: C-X-C chemokine receptor
CYLD: cylindromatosis
CYP2E1: cytochrome P450 2E1
DPP-4: dipeptidyl peptidase-4
EPA: eicosapentaenoic acid
ER: endoplasmic reticulum
ERK: extracellular signal-regulated kinase)
FAP: fibroblast activation protein
FcγRIIA: low-affinity immunoglobulin gamma Fc region receptor II-a
FSTL1: follistatin-like protein 1
IFNAR-1: interferon alpha/beta receptor subunit 1
FOXO1: forkhead box O1
GLAST: glutamate-aspartate transporter
GLP-1RA: glucagon-like peptide-1 receptor agonist
GLUT1: glucose transporter 1
HCC: hepatocellular carcinoma
HGF: hepatocyte growth factor
HMGB1: high mobility group box 1
HNF4alpha: hepatocyte nuclear factor 4 alpha
HRG: histidine-rich glycoprotein
HSC: hepatic stellate cell
HSPA5: heat shock protein family A member 5
IGF-1: insulin-like growth factor I
IL: interleukin
iNOS: inducible nitric oxide synthase
IRF5: interferon regulatory factor 5
JAK: Janus kinase
KCs: Kupffer cells
KLF4: krüppel-like factor 4
Kv1.3: voltage-gated potassium channel 1.3
LAMs: lipid-associated macrophages
LPS: lipopolysaccharide
Ly6C: lymphocyte antigen 6 complex, locus C
M1: classically activated macrophage phenotype
M2: alternatively activated macrophage phenotype
MASH: metabolic dysfunction-associated steatohepatitis
MASLD: metabolic dysfunction-associated steatotic liver disease
MERTK: MER tyrosine kinase
MLL4: mixed-lineage leukemia protein 4
MMP: matrix metalloproteinase
Mo-KCs: monocyte-derived macrophages that repopulate the Kupffer cell niche
MSCs: mesenchymal stem/stromal cells
mtDNA: mitochondrial DNA
mTOR: mechanistic target of rapamycin
MyD88: myeloid differentiation primary response 88
NASH: non-alcoholic steatohepatitis
NF-κB: negative regulator of nuclear factor-κB
NFLD: Non-Alcoholic Fatty Liver Disease
NOTCH2: notch receptor 2
NRF2: nuclear factor erythroid 2–related Factor 2
PGC-1α: peroxisome proliferator-activated receptor gamma coactivator-1α
PKM2: pyruvate kinase M2
PPAR: peroxisome proliferator–activated receptor
PSTPIP2: proline-serine-threonine phosphatase-interacting protein 2
RBP-J: recombination signal binding protein for immunoglobulin κ
RCT: randomized clinical trial
RNF41: ring finger protein 41
RORα: orphan nuclear receptor retinoic acid–related orphan receptor α
ROS: reactive oxygen species
SGLT2: sodium–glucose cotransporter 2
SGLT2i: SGLT2 inhibitor
SIRT: sirtuin
SOCS1: suppressor of cytokine signaling 1
SphK1: sphingosine kinase 1
STAT: signal transducer and activator of transcription
STING: stimulator of interferon genes
T2DM: type-2 *diabetes mellitus;* TCA, tricarboxylic acid
TCM: traditional Chinese medicinal
TGF-β1: transforming growth factor-β1
TLR: toll-like receptor
TM4SF5: transmembrane 4 L six family member 5
TREM: triggering receptor expressed on myeloid cells
TRIM38: tripartite motif-containing protein 38
TRPM2: transient receptor potential melastatin 2
VEGF: vascular endothelial growth factor
Wnt1: wingless-type MMTV integration site family, member 1
